# Estimation methods for the variance of Birnbaum-Saunders distribution containing zero values with application to wind speed data in Thailand

**DOI:** 10.7717/peerj.18272

**Published:** 2024-10-16

**Authors:** Natchaya Ratasukharom, Sa-Aat Niwitpong, Suparat Niwitpong

**Affiliations:** Department of Applied Statistics, King Mongkut’s University of Technology North Bangkok, Bangkok, Thailand

**Keywords:** Particulate matter, Confidence interval, Generalized confidence interval, Bootstrap confidence interval, Generalized fiducial confidence interval, Normal approximation, Simulation

## Abstract

Thailand is currently grappling with a severe problem of air pollution, especially from small particulate matter (PM), which poses considerable threats to public health. The speed of the wind is pivotal in spreading these harmful particles across the atmosphere. Given the inherently unpredictable wind speed behavior, our focus lies in establishing the confidence interval (CI) for the variance of wind speed data. To achieve this, we will employ the delta-Birnbaum-Saunders (delta-BirSau) distribution. This statistical model allows for analyzing wind speed data and offers valuable insights into its variability and potential implications for air quality. The intervals are derived from ten different methods: generalized confidence interval (GCI), bootstrap confidence interval (BCI), generalized fiducial confidence interval (GFCI), and normal approximation (NA). Specifically, we apply GCI, BCI, and GFCI while considering the estimation of the proportion of zeros using the variance stabilized transformation (VST), Wilson, and Hannig methods. To evaluate the performance of these methods, we conduct a simulation study using Monte Carlo simulations in the R statistical software. The study assesses the coverage probabilities and average widths of the proposed confidence intervals. The simulation results reveal that GFCI based on the Wilson method is optimal for small sample sizes, GFCI based on the Hannig method excels for medium sample sizes, and GFCI based on the VST method stands out for large sample sizes. To further validate the practical application of these methods, we employ daily wind speed data from an industrial area in Prachin Buri and Rayong provinces, Thailand.

## Introduction

Every year from November to April, Thailand experiences elevated particulate matter with a diameter of fewer than 2.5 microns (PM2.5) levels due to fluctuating weather conditions, particularly during the dry to winter season (Amnuaylojaroen et al., 2020; Fold et al., 2020). During this period, PM2.5 concentrations consistently exceed the standards set by the World Health Organization (WHO). This period is characterized by high air pressure, a cloudless sky, and calm weather, which lead to the accumulation of pollutants and, subsequently, elevated pollution levels. Additionally, combustion from engines, agricultural activities, and industrial pollution exacerbate the problem, resulting in higher-than-normal pollution levels during these months. PM2.5 poses a significant health risk, especially for vulnerable populations (Suebyart & Imdee, 2023) such as children, pregnant women, allergy sufferers, and the elderly. These tiny particles, smaller than 2.5 microns, easily bypass the nasal hair filtration system, travel through the respiratory tract, and penetrate the capillaries, ultimately entering the bloodstream. This process can lead to various respiratory diseases, underscoring the importance of addressing and mitigating PM2.5 pollution. Paneangtong, MaleeHuan & Chamchoa (2012) state that individuals residing in industrial areas often experience more health problems from PM2.5 pollution than those living outside these areas. In 2023, Prachin Buri and Rayong provinces, located in an industrial area, recorded their highest level at 81 micrograms per cubic meter, resulting in an air quality index (AQI) of 207, categorized as very unhealthy (Pollution Control Department, http://air4thai.pcd.go.th/webV3/#/History). This severe classification highlights the significant impact on public health in such regions. Amnuaylojaroen, Kaewkanchanawong & Panpeng (2023) found an inverse relationship between wind speed and PM2.5, suggesting that increasing wind speed can help reduce PM2.5 levels. Additionally, Mohammadi, Alavi & McGowan (2017) explored the use of the two-parameter Birnbaum-Saunders (BirSau) distribution to analyze wind speed and wind power density across ten stations in Canada. Their findings demonstrate that the BirSau distribution performed exceptionally well across all selected stations. Therefore, estimating wind speed using the BirSau distribution is of interest. However, the BirSau distribution is suitable for data with values greater than zero. When considering wind speed, we find that it fluctuates daily due to natural variations, which may result in values being either zero and greater than zero. Hence, it is recommended to use the zero-inflated Birnbaum-Saunders distribution instead.

The zero-inflated Birnbaum-Saunders distribution, also known as the delta-Birnbaum-Saunders distribution (delta-BirSau), is a distribution that has yet to be explored in previous research. The delta-BirSau distribution is designed for non-negative data that includes zero values with a probability $(0 \;< \; \delta \;< \; 1)$. It integrates BirSau distributions with shape parameter $(\alpha )$ and scale parameter $(\beta )$, encompassing positive values with the remaining probability $(1 - \delta )$, and zero observations following a binomial distribution with a binomial proportion $(\delta )$, based on the concept introduced by Aitchison (1955). This concept has been extensively applied in various other distributions, including the delta-lognormal (Wu & Hsieh, 2014; Fletcher, 2008) and delta-gamma (Muralidharan & Kale, 2002; Kaewprasert, Nitwitpong & Nitwitpong, 2022) distributions. So, we conceived the idea of integrating this concept with the BirSau distribution. For the BirSau distribution, initially proposed by Birnbaum & Saunders (1969a), the BirSau distribution was introduced as a model for the time until failure. It is designed to characterize the overall duration until a component fails due to the development and expansion of a primary crack, ultimately reaching a specified damage threshold. The BirSau distribution has applications in various fields, including finance, engineering, and the environment. However, it may need to be better suited for handling data equal to zero. Addressing this limitation, the delta-Birsau distribution serves as a solution, allowing for the utilization of the distribution with both positive data and data equal to zero.

Many researchers are interested in estimating the parameters of a BirSau distribution. First, the maximum likelihood estimators (MLEs) of the parameters $\alpha$ and $\beta$ were derived by Birnbaum & Saunders (1969b). According to Balakrishnan et al. (2009), moment estimators may only sometimes exist in all of these cases, and explicit expressions for these estimators are impossible to find, necessitating numerical approaches. For this reason, to estimate the parameters $\alpha$ and $\beta$, Ng, Kundu & Balakrishnan (2003) introduced modified moment estimators (MMEs) and then applied a bias-reduction technique to mitigate bias in their MLEs and MMEs. Lemonte, Cribari-Neto & Vasconcellos (2007) also developed MMEs for the parameters $\alpha$ and $\beta$. Sun (2009) introduced the generalized confidence interval specifically for the parameter $\beta$. Xu & Tang (2011) introduced a Bayesian analysis of BirSau distribution with partial information. Subsequently, Wang (2012) extended this methodology by proposing the generalized confidence interval for the parameter $\alpha$. Li & Xu (2016) introduced a method of fiducial inference designed explicitly for estimating the parameters of the BirSau distribution. Furthermore, Lemonte, Simas & Neto (2008) presented several strategies for correcting bias in the MLEs of parameters $\alpha$ and they were using the bootstrap method. Their investigation revealed that employing the concept of constant-bias-correcting (CBC) estimates, as proposed by Mackinnon & Smith (1998), proved to be the most effective method for reducing bias. Next, Vilca, Zeller & Balakrishnan (2023) proposed a multivariate generalization of BirSau distribution based on the multivariate skew-normal distribution, presenting distributional properties and an EM algorithm for parameter estimation. Lastly, Xu et al. (2024) studied the Bayesian method to analyze degradation data with small sample sizes. Many researchers have developed various methods to estimate the parameter $\delta$ with a binomial distribution. For example, for creating intervals of $\delta$, Wu & Hsieh (2014) employed the generalized pivotal quantity (GPQ) based on the variance stabilized transformation (VST). Wilson (1927) suggested using the Wilson interval for $\delta$ in an interval approach. Hannig, Iyer & Patterson (2006) presented the idea for the generalized fiducial quantity (GFQ) by combining two beta distributions to estimate the parameter $\delta$.

The variance is a measure of interest in statistical inference that describes the deviation from the average (mean). The variance is defined as the second central moment, and the standard deviation is the positive square root of the variance (Casella & Berger, 2002). Furthermore, Casella & Berger (2002) argue that confidence intervals are more effective in capturing the parameter of interest when compared to point estimates. Confidence intervals have been utilized to estimate the variability, specifically the variance, of several distributions. For example, Krishnamoorthy, Mathew & Ramachandran (2006) applied the idea of GCI to construct the confidence intervals for the variance of lognormal distribution. Cojbasic & Tomovic (2007) introduced confidence intervals for both the variance and the difference in variances of an exponential distribution. These intervals are derived using ordinary t-statistics in conjunction with the bootstrap method. Panichkitkosolkul (2013) conducted a study on a double bootstrap-t one-sided confidence interval for the population variance of skewed distributions. Paksaranuwat & Niwitpong (2015) compare confidence intervals for the variance and the ratio of two variances in cases where the population distributions are non-normal, and item nonresponse is present. This comparison involves utilizing classical and adaptive confidence intervals based on the Bonett method. Thangjai, Niwitpong & Niwitpong (2018) introduced confidence intervals for the single variance and the difference between two exponential population variances. This was accomplished using a generalized confidence interval, a large sample confidence interval, and an exact confidence interval. Maneerat, Niwitpong & Niwitpong (2021) suggest a variational approximation using an interval estimator derived from a Bayesian methodology for the variance of the delta-lognormal distribution. This method incorporates the highest posterior density interval based on a vague prior (HPD-V), and the variance estimates recovery method (MOVER). Araveeporn (2022) compared the estimation of confidence intervals for the variance of a normal distribution using maximum likelihood, the Chi-squared distribution, and the Bayesian credible interval. Puggard, Niwitpong & Niwitpong (2022a) presented confidence intervals for the variance and the difference of variances of BirSau distributions. They found that the bootstrap confidence interval (BCI) outperformed the other confidence intervals in terms of average length, and the generalized confidence interval (GCI) had coverage probability (CP) greater than or close to the nominal confidence level. In later studies, Puggard, Niwitpong & Niwitpong (2022b) examined the estimation of the confidence interval for the variance ratio of the BirSau distribution and found that the generalized fiducial confidence interval (GFCI) had CP greater than or close to the nominal confidence level. Therefore, we are interested in using these methods to construct confidence intervals for the parameters $\alpha$ and $\beta$. Specifically, methods like GCI, BCI, and GFCI are designed to estimate wind speed data, as they are well-suited for fitting the BirSau distribution, which is suitable for modeling wind speed. Hence, GCI, BCI, and GFCI methods are appropriate for estimating wind speed data. Maneerat, Nakjai & Niwitpong (2022) investigated the estimation of the confidence interval for the ratio of medians using the wind speed data and found that the normal approximation (NA) method has CP values greater than or close to the nominal confidence level. Therefore, there is also an interest in using the NA method for estimation. Hence, we are interested in using the GCI, BCI, GFCI, and NA methods to estimate the parameters of the delta-BirSau distribution.

Nevertheless, the construction of the confidence interval for the variance of the delta-BirSau distribution has yet to be studied. Therefore, the objective of this study is to propose ten methods for constructing intervals for the variance of the delta-BirSau distribution. These methods are based on the GCI, BCI, GFCI, and NA. Specifically, GCI, BCI, and GFCI are employed, considering the estimation of $\delta$ using the VST, Wilson, and Hannig methods. The effectiveness of the proposed confidence intervals was evaluated through Monte Carlo simulations to assess their CPs and averaged widths (AWs). We also demonstrated their practical utility by applying them to estimate daily wind speed data from an industrial area in Prachin Buri and Rayong provinces, Thailand, from October to December 2023.

## Methods

**Figure 1:**
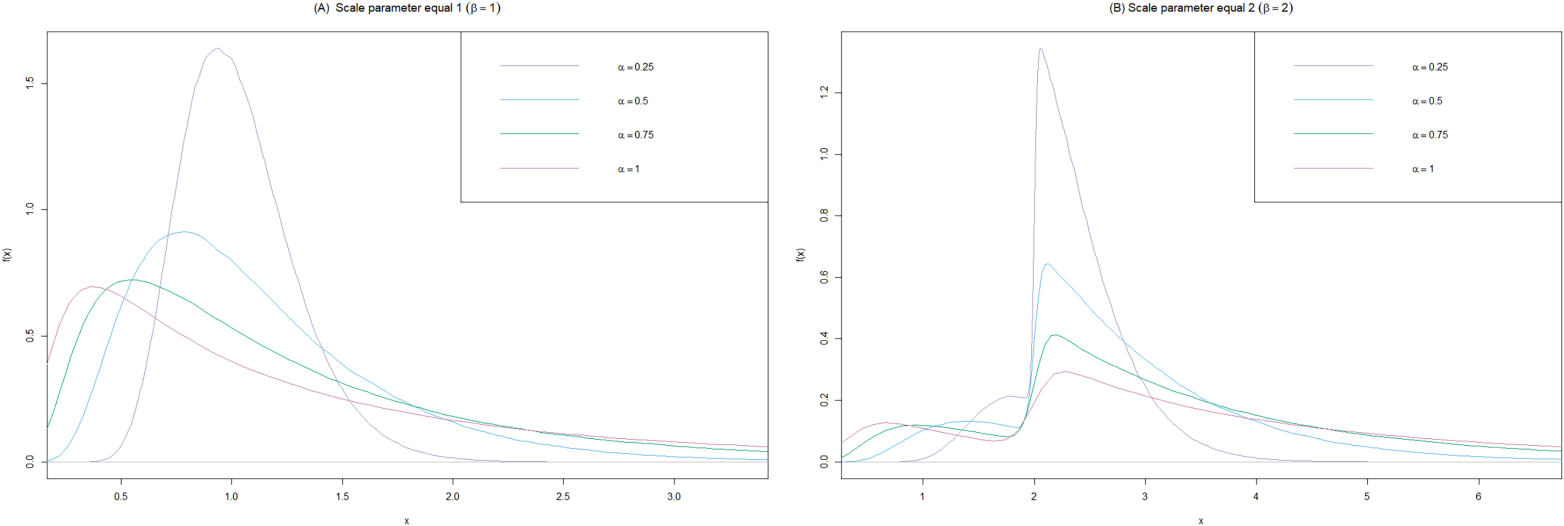
Comparison of the BirSau for some values. (A) Scale parameter equal to 1 and (B) scale parameter equal to 2.

Let $X = ({X_1},{X_2},...,{X_n})$ be a random sample from the delta-BirSau distribution, denoted as $ \Delta (\alpha ,\beta ,\delta )$. The distribution function of $X$ is introduced by Aitchison (1955), and Birnbaum & Saunders (1969a) is defined as

(1) $$G(x;\,\alpha ,\beta ,\,\delta )\, = \,\left\{ \matrix{
  \delta ;\,\,\,\,\,\,\,\,\,\,\,\,\,\,\,\,\,\,\,\,\,\,\,\,\,\,\,\,\,\,\,\,\,\,\,\,\,\,\,\,\,\,\,\,\,\,\,\,\,\,\,\,\,\,\,\,\,\,\,\,\,\,x = 0 \hfill \cr 
  \,\delta  + \,(1\,-\,\delta )F(x;\,\alpha ,\beta );\,\,\,\,\,\,\,x > 0 \hfill \cr}  \right.\,\,,$$

where $\delta = P(x = 0)$ follow the binomial distribution and ${n_{(0)}} \sim bi(n,\delta )$ and $F(X;\alpha ,\beta )$ is denoted as BirSau distribution. The density of the BirSau distribution is skewed to the right. The shape parameter $\left( \alpha \right)$ affects the slope of the function, with the asymmetry decreasing as $\alpha$ increases. The scale parameter $\left( \beta \right)$ determines the spread of the distribution while $\delta$ indicates the proportion of zero values. If the $\delta$ increases, it increases zero values. The BirSau density for some values $\alpha$ with $\beta$ = 1, and 2 is shown in Fig. 1.

The maximum likelihood estimator of $\delta$ is $\hat \delta = {n_{(0)}}/n$; where ${n_{(1)}}$ and ${n_{(0)}}$ are the number of positive observed values and the number of zero, respectively, and $n = {n_{(0)}} + {n_{(1)}}$. The population mean and variance of $X$ are respectively given by

(2) $$E(X) = (1 - \delta )(\beta )\left( {1 + \displaystyle{{{\alpha ^2}} \over 2}} \right),$$

(3) $$Var(X) = (1 - \delta )({\beta ^2})\left[ {{\alpha ^2}\left( {1 + \displaystyle{5 \over 4}{\alpha ^2}} \right) + \delta {{\left( {1 + \displaystyle{1 \over 2}{\alpha ^2}} \right)}^2}} \right].$$

In this study, our interest was in estimating the confidence interval of the variance, which is defined as $\tau$, as shown below:

(4) $$\tau = (1 - \delta )({\beta ^2})\left[ {{\alpha ^2}\left( {1 + \displaystyle{5 \over 4}{\alpha ^2}} \right) + \delta {{\left( {1 + \displaystyle{1 \over 2}{\alpha ^2}} \right)}^2}} \right].$$

The following subsection provides a detailed explanation of the approaches utilized for constructing the confidence intervals.

### Generalized confidence interval: GCI

Weerahandi (1993) originated the idea of the GPQ for deriving confidence intervals. The GPQ comprises two key concepts. Firstly, the GPQ has a distribution free of unknown parameters of interest. Secondly, the observed value of the GPQ does not depend on nuisance parameters.

Let ${X} = {X_1},{X_2},...,{X_{{n_{(1)}}}}$ be a random sample size ${n_{(1)}}$ from $BirSau(\alpha ,\beta )$ distribution. First, let $\alpha$ be the parameter of interest, and $\beta$ be a nuisance parameter. The GPQs of $\beta$ were proposed by Sun (2009) and are defined as follows:

(5) $$O_\beta ^{GCI}: = O_\beta ^{GCI}(x;\kappa ) = \left\{ \matrix{\max ({\beta _{1,}}{\beta _2}),\,\,\,\,\,\kappa \le 0;  \cr \min ({\beta _{1,}}{\beta _2}),\,\,\,\,\,\,\kappa \;> \; 0. } \right.$$

The GPQs of $\alpha$ introduced by Wang (2012) is obtained as

(6) $$O_\alpha ^{GCI}: = O_\alpha ^{GCI}({x},\nu ,\kappa ) = {\left[ {\displaystyle{{{s_2}{{\left( {O_\beta ^{GCI}} \right)}^2} - 2{n_{(1)}}O_\beta ^{GCI} + {s_1}} \over {O_\beta ^{GCI}\omega }}} \right]^{{1 / 2}}},$$

where $x = ({x_1},{x_2},...,{x_{{n_{(1)}}}})$ are the observed values of $X$, $\kappa$ has a *t*-distribution with ${n_{(1)}} - 1$ degrees of freedom $\left( {\kappa \sim t({n_{(1)}} - 1)} \right)$, ${s_1} = \sum\nolimits_{i = 1}^{{n_{(1)}}} {{X_i}}$, ${s_2} = \sum\nolimits_{i = 1}^{{n_{(1)}}} {{1 \mathord{\left/ {\vphantom {1 {{X_i}}}} \right.} {{X_i}}}}$ and $\omega$ has a chi-squared distribution with ${n_{(1)}}$ degrees of freedom $\left( {\omega \sim {\chi ^2}_{{n_{(1)}}}} \right)$.

From Eq. (6), ${\beta _1}$ and ${\beta _2}$ are the two solutions of the following equation:

(7) $$ \left[({n_{(1)}} - 1){Q^2} - \displaystyle{1 \over {{n_{(1)}}}}S{\kappa ^2}\right] {\beta ^2} - 2[({n_{(1)}} - 1)PQ - (1 - PQ){\kappa ^2}]\beta + ({n_{(1)}} - 1){P^2} - \displaystyle{1 \over {{n_{(1)}}}}R{\kappa ^2} = 0,$$

where $P = n_{(1)}^{ - 1}\sum\nolimits_{i = 1}^{{n_{(1)}}} {\sqrt {{X_i}} }$, $Q = n_{(1)}^{ - 1}\sum\nolimits_{i = 1}^{{n_{(1)}}} {1/\sqrt {{X_i}} }$, $R = \sum\nolimits_{i = 1}^{{n_{(1)}}} {{{(\sqrt {{X_i}} - P)}^2}}$, and $S = \sum\nolimits_{i = 1}^{{n_{(1)}}} {{{(1/\sqrt {{X_i}} - Q)}^2}}$.

#### The generalized confidence interval based on the VST method (GCI-1)

Dasgupta (2008) introduced the VST method, under normal approximation. Wu & Hsieh (2014) derived the VST for a binomial distribution, approximating it with the arcsine square-root transformation. Consequently, the GPQ for $\delta$, as proposed by Wu & Hsieh (2014), is expressed as follows

(8) $$O_\delta ^{VST} = {\sin ^2} \left(\arcsin \sqrt {\hat \delta } - \displaystyle{T \over {2\sqrt n }}\right),$$

where $T = 2\sqrt n (\arcsin \sqrt {\hat \delta } - \arcsin \sqrt \delta ) \sim N(0,1);n \to \infty$. Hence, through the use of three pivots of $O_\beta ^{GCI}$, $O_\alpha ^{GCI}$, and $O_\delta ^{VST}$ denoted as Eqs. (5), (6), and (8), respectively, the GPQ for $\tau$ is defined as

**Algorithm 1:** GCI

1. Define the parameters $\alpha$, $\beta$, and $\delta$.
2. Define $n$, ${n_{\left( 0 \right)}}$, and ${n_{\left( 1 \right)}}$, where ${n_{\left( 0 \right)}}$ is determined as a realization from ${n_{(0)}} \sim bi(n,\delta )$, and ${n_{\left( 1 \right)}}$
is calculated as ${n_{\left( 1 \right)}} = n - {n_{\left( 0 \right)}}$.
3. Simulate the datasets using the delta-BirSau distribution with parameters $\alpha$, $\beta$, and $\delta$, where
$X\sim{\rm delta - BirSau}(\alpha ,\beta ,\delta )$.
4. Consider datasets containing values greater than zero.
5. Calculate $P,Q,R,S,{s_1}$ and ${s_2}$, respectively.
6. During the ${i^{th}}$ iteration:
(a) Generate $\kappa \sim t({n_{(1)}} - 1)$ to calculate the generalized confidence interval of $\alpha$ denote as
$O_\alpha ^{GCI} \;> \;0$ by using Eq. (5).
(b) Generate $\omega \sim {\chi ^2}_{{n_{(1)}}}$, then calculate the generalized confidence interval of $\beta$ denote as
$O_\beta ^{GCI}$ by using Eq. (6).
Perform step 6 interactively for a total of $I$ replications.

(9) $$O_\tau ^{GCI.VST} = (1 - O_\delta ^{VST}){(O_\beta ^{GCI})^2}\left( {{{\left( {O_\alpha ^{GCI}} \right)}^2}\left( {1 + \displaystyle{5 \over 4}{{\left( {O_\alpha ^{GCI}} \right)}^2}} \right) + O_\delta ^{VST}{{\left( {1 + \displaystyle{1 \over 2}{{\left( {O_\alpha ^{GCI}} \right)}^2}} \right)}^2}} \right).$$

Therefore, the $100(1 - \gamma )\%$ confidence interval for $\tau$ using the VST method for GCI is

(10) $$CI_\tau ^{GCI.VST} = \,[O_\tau ^{GCI.VST}(\gamma /2),O_\tau ^{GCI.VST}(1 - \gamma /2)],$$

where $O_\tau ^{GCI.VST}(\gamma /2)$ and $O_\tau ^{GCI.VST}(1 - \gamma /2)$ are the $100{(\gamma /2)^{th}}$ and $100{(1 - \gamma /2)^{th}}$ percentiles of the distribution of $O_\tau ^{GCI.VST}$, respectively.

#### The generalized confidence interval based on the Wilson score method (GCI-2)

Using the score interval defined by Wilson (1927), Li, Zhou & Tian (2013) established the GPQ for $\delta$ for a binomial distribution as follows

(11) $$O_\delta ^W = \displaystyle{{{n_{(0)}} + Z_W^2/2} \over {n + Z_W^2}} - \displaystyle{{{Z_W}} \over {n - Z_W^2}}{\left[ {{n_{(0)}}\left( {1 - \displaystyle{{{n_{(0)}}} \over n}} \right) + \left. {\displaystyle{{Z_W^2} \over 4}} \right]} \right.^{1/2}},$$

where ${Z_W}$ has a standard normal distribution. Therefore, the GPQ of $\tau$ is defined by the three pivots of $O_\beta ^{GCI}$, $O_\alpha ^{GCI}$, and $O_\delta ^W$, in Eqs. (5), (6), and (11) as

(12) $$O_\tau ^{GCI.W} = (1 - O_\delta ^W){(O_\beta ^{GCI})^2}\left( {{{\left( {O_\alpha ^{GCI}} \right)}^2}\left( {1 + \displaystyle{5 \over 4}{{\left( {O_\alpha ^{GCI}} \right)}^2}} \right) + O_\delta ^W{{\left( {1 + \displaystyle{1 \over 2}{{\left( {O_\alpha ^{GCI}} \right)}^2}} \right)}^2}} \right).$$

Therefore, the $100(1 - \gamma )\%$ confidence interval for $\tau$ using the Wilson method for GCI is

(13) $$CI_\tau ^{GCI.W} = \,[O_\tau ^{GCI.W}(\gamma /2),O_\tau ^{GCI.W}(1 - \gamma /2)],$$

where $O_\tau ^{GCI.W}(\gamma /2)$ and $O_\tau ^{GCI.W}(1 - \gamma /2)$ are the $100{(\gamma /2)^{th}}$ and $100{(1 - \gamma /2)^{th}}$ percentiles of the distribution of $O_\tau ^{GCI.W}$, respectively.

#### The generalized confidence interval based on the Hannig method (GCI-3)

Hannig (2009) introduced the GFQ of $\delta$. According to a previous study, the optimal GFQ for $\delta$ is the product of two beta distributions, each weighted by 0.5. Subsequently, the investigation by Li, Zhou & Tian (2013) also utilized the previously reported GFQ. Furthermore, it aligns with the GPQ concept. As a consequence, this study employs the GPQ for $\delta$, which is

(14) $$O_\delta ^H \sim 0.5beta({n_{(0)}},{n_{(1)}} + 1) + 0.5beta({n_{(0)}} + 1,{n_{(1)}}).$$

From $O_\beta ^{GCI}$, $O_\alpha ^{GCI}$, and $O_\delta ^H$ in Eqs. (5), (6), and (14), the approximate GPQ of $\tau$ was

(15) $$O_\tau ^{GCI.H} = (1 - O_\delta ^H){(O_\beta ^{GCI})^2}\left( {{{\left( {O_\alpha ^{GCI}} \right)}^2}\left( {1 + \displaystyle{5 \over 4}{{\left( {O_\alpha ^{GCI}} \right)}^2}} \right) + O_\delta ^H{{\left( {1 + \displaystyle{1 \over 2}{{\left( {O_\alpha ^{GCI}} \right)}^2}} \right)}^2}} \right).$$

Therefore, the $100(1 - \gamma )\%$ confidence interval for $\tau$ using Hannig method for GCI is

(16) $$CI_\tau ^{GCI.H} = \,[O_\tau ^{GCI.H}(\gamma /2),O_\tau ^{GCI.H}(1 - \gamma /2)],$$

where $O_\tau ^{GCI.H}(\gamma /2)$ and $O_\tau ^{GCI.H}(1 - \gamma /2)$ are the $100{(\gamma /2)^{th}}$ and $100{(1 - \gamma /2)^{th}}$ percentiles of the distribution of $O_\tau ^{GCI.H}$, respectively.

### Bootstrap confidence interval: BCI

Bootstrap methods were developed by Efron & Tibishirani (1993) using resampling techniques. Bootstrap estimators for $\alpha$ and $\beta$ in BirSau distributions are calculated using the methods proposed by Lemonte, Simas & Neto (2008) to reduce bias in the MLE. The method is outlined as follows:

Let ${\bf x} = {({x_1},{x_2},...,{x_{{n_{(1)}}}})^T}$ be a random sample of size ${n_{(1)}}$, from the random variable ${X}$ with the distribution function ${F_\alpha }(x)$ and ${F_\beta }(x)$ and $\hat \alpha ,\hat \beta$ be an estimator of $\alpha ,\beta$ base on $x$. The $B$ bootstrap sample $\left( {{{\rm x}^{{\rm *1}}}{\rm ,}{{\rm x}^{{\rm *2}}}{\rm ,}...{\rm ,}{{\rm x}^{{\rm *}B}}} \right)$ are generated from the original sample $x$. The respective bootstrap replications for $\alpha$ and $\beta$ are denoted as $({\hat \alpha ^{*1}},{\hat \alpha ^{*2}},...,{\hat \alpha ^{*B}})$ and $({\hat \beta ^{*1}},{\hat \beta ^{*2}},...,{\hat \beta ^{*B}})$, where ${\hat \alpha ^{*b}} = s({{\bf x}^{*b}}),$ ${\hat \beta ^{*b}} = s({{\bf x}^{*b}})$ and $b = 1,2,...,B$. The approximated bootstrap of $\alpha$ and $\beta$ are calculated by ${\hat \alpha ^{*(.)}} = (\sum\nolimits_{b = 1}^B {{{\hat \alpha }^{*b}}} )/B$ and ${\hat \beta ^{*(.)}} = (\sum\nolimits_{b = 1}^B {{{\hat \beta }^{*b}}} )/B$. Therefore, the bootstrap bias is estimated based on $B$ replications of the $\hat \alpha$ and $\hat \beta$ are ${\hat B_{{F_{\hat \alpha }}}}(\hat \alpha ,\alpha ) = {\hat \alpha ^{*(.)}} - \hat \alpha$ and ${\hat B_{{F_{\hat \beta }}}}(\hat \beta ,\beta ) = {\hat \beta ^{*(.)}} - \hat \beta$. Then, the CBC method, invented by Mackinnon & Smith (1998), is applied to reduce the bias value of the estimator. Subsequently, the estimate with reduced bias value is obtained as follows:

(17) $$O_\alpha ^{BCI} = \hat \alpha - {\hat B_{{F_{\hat \alpha }}}}(\hat \alpha ,\alpha ) = 2\hat \alpha - {\hat \alpha ^{*(.)}},$$

and

(18) $$O_\beta ^{BCI} = \hat \beta - {\hat B_{{F_{\hat \beta }}}}(\hat \beta ,\beta ) = 2\hat \beta - {\hat \beta ^{*(.)}}.$$

**Algorithm 2:** BCI

1. Define the parameters $\alpha$, $\beta$, and $\delta$.
2. Define $n$, ${n_{\left( 0 \right)}}$, and ${n_{\left( 1 \right)}}$, where ${n_{\left( 0 \right)}}$ is determined as a realization from ${n_{(0)}} \sim bi(n,\delta )$, and ${n_{\left( 1 \right)}}$
is calculated as ${n_{\left( 1 \right)}} = n - {n_{\left( 0 \right)}}$.
3. Simulate the datasets using the delta-BirSau distribution with parameters $\alpha$, $\beta$, and $\delta$, where
$X\sim{\rm delta - BirSau}(\alpha ,\beta ,\delta )$.
4. Consider datasets containing values greater than zero.
5. At $B$ step:
(a) Generate ${{\bf x}^{\rm *}} = \left( {{{\rm x}^{{\rm *1}}}{\rm ,}{{\rm x}^{{\rm *2}}}{\rm ,}...{\rm ,}{{\rm x}^{{\rm *}B}}} \right)$ with replacement from ${\bf x}{\rm = }{({x_1},{x_2},...,{x_{{n_{(1)}}}})^T}$.
(b) Compute the bias of the estimator of $\hat \alpha$ and $\hat \beta$, which are ${\hat B_{{F_{\hat \alpha }}}}(\hat \alpha ,\alpha ) = {\hat \alpha ^{*(.)}} - \hat \alpha$ and
${\hat B_{{F_{\hat \beta }}}}(\hat \beta ,\beta ) = {\hat \beta ^{*(.)}} - \hat \beta ,$ respectively.
(c) Compute the correct estimator of $\hat \alpha$ and $\hat \beta$ are $O_\alpha ^{BCI}$ and $O_\beta ^{BCI}$ using Eqs. (17) and (18),
respectively.
Repeat step 5, total of $B$ times.

#### The bootstrap confidence interval based on the VST method (BCI-1)

By utilizing $O_\delta ^{VST}$ in Eq. (8) and the bootstrap estimators of $O_\alpha ^{BCI}$ and $O_\beta ^{BCI}$ in Eqs. (17) and (18), the bootstrap estimator of $\tau$ based on the VST method can be written as:

(19) $$O_\tau ^{BCI.VST} = (1 - O_\delta ^{VST}){(O_\beta ^{BCI})^2}\left( {{{\left( {O_\alpha ^{BCI}} \right)}^2}\left( {1 + \displaystyle{5 \over 4}{{\left( {O_\alpha ^{BCI}} \right)}^2}} \right) + O_\delta ^{VST}{{\left( {1 + \displaystyle{1 \over 2}{{\left( {O_\alpha ^{BCI}} \right)}^2}} \right)}^2}} \right).$$

Therefore, the $100(1 - \gamma )\%$ confidence interval for $\tau$ using the VST method for BCI is

(20) $$CI_\tau ^{BCI.VST} = \,[O_\tau ^{BCI.VST}(\gamma /2),O_\tau ^{BCI.VST}(1 - \gamma /2)],$$

where $O_\tau ^{BCI.VST}(\gamma /2)$ and $O_\tau ^{BCI.VST}(1 - \gamma /2)$ are the $100{(\gamma /2)^{th}}$ and $100{(1 - \gamma /2)^{th}}$ percentiles of the distribution of $O_\tau ^{BCI.VST}$, respectively.

#### The bootstrap confidence interval based on the Wilson score method (BCI-2)

By utilizing Eq. (11) with $O_\delta ^W$, along with the bootstrap estimator of $O_\alpha ^{BCI}$ and $O_\beta ^{BCI}$ presented in Eqs. (17) and (18), the bootstrap estimator based on the Wilson score method can be expressed as:

(21) $$O_\tau ^{BCI.W} = (1 - O_\delta ^W){(O_\beta ^{BCI})^2}\left( {{{\left( {O_\alpha ^{BCI}} \right)}^2}\left( {1 + \displaystyle{5 \over 4}{{\left( {O_\alpha ^{BCI}} \right)}^2}} \right) + O_\delta ^W{{\left( {1 + \displaystyle{1 \over 2}{{\left( {O_\alpha ^{BCI}} \right)}^2}} \right)}^2}} \right).$$

Therefore, the $100(1 - \gamma )\%$ confidence interval for $\tau$ using the Wilson score method for BCI is

(22) $$CI_\tau ^{BCI.W} = \,[O_\tau ^{BCI.W}(\gamma /2),O_\tau ^{BCI.W}(1 - \gamma /2)],$$

where $O_\tau ^{BCI.W}(\gamma /2)$ and $O_\tau ^{BCI.W}(1 - \gamma /2)$ are the $100{(\gamma /2)^{th}}$ and $100{(1 - \gamma /2)^{th}}$ percentiles of the distribution of $O_\tau ^{BCI.W}$, respectively.

#### The bootstrap confidence interval based on the Hannig method (BCI-3)

By incorporating $O_\delta ^H$ in Eq. (14), along with the bootstrap estimator of $O_\alpha ^{BCI}$ and $O_\beta ^{BCI}$ in Eqs. (17) and (18), the bootstrap estimator based on the Hannig method can be formulated as follows:

(23) $$O_\tau ^{BCI.H} = (1 - O_\delta ^H){(O_\beta ^{BCI})^2}\left( {{{\left( {O_\alpha ^{BCI}} \right)}^2}\left( {1 + \displaystyle{5 \over 4}{{\left( {O_\alpha ^{BCI}} \right)}^2}} \right) + O_\delta ^H{{\left( {1 + \displaystyle{1 \over 2}{{\left( {O_\alpha ^{BCI}} \right)}^2}} \right)}^2}} \right).$$

Therefore, the $100(1 - \gamma )\%$ confidence interval for $\tau$ using the Hannig method for BCI is

(24) $$CI_\tau ^{BCI.H} = \,[O_\tau ^{BCI.H}(\gamma /2),O_\tau ^{BCI.H}(1 - \gamma /2)],$$

where $O_\tau ^{BCI.H}(\gamma /2)$ and $O_\tau ^{BCI.H}(1 - \gamma /2)$ are the $100{(\gamma /2)^{th}}$ and $100{(1 - \gamma /2)^{th}}$ percentiles of the distribution of $O_\tau ^{BCI.H}$, respectively.

### Generalized fiducial confidence interval: GFCI

Generalized fiducial inference allows for converting the original data into known alternative distributions. Subsequently, based on the outcomes derived from these distributions, the transformed data are manipulated, and the results are then reverted to the original form through an inverse transformation (Hannig, 2009).

Let ${W}$ be a vector randomly distributed, with its index determined by the parameter ${\xi }\, \in \,\Xi$. It is important to note that the procedure for generating the data for ${U}$ can be described as follows:

(25) $${U} = {V}({\xi ,W}),$$

where ${V}$ is a jointly measurable function and ${W}$ is a random variable or random vector with a completely known distribution and independent of any parameter. The inverse will be written as ${Q}\left( {{U,W}} \right)$, we can calculate ${\xi }$ from ${u} = {Q}({\xi }{,w})$. Since the distribution of ${W}$ is completely known, a random sample ${{w}_1},{{w}_2}, \ldots ,{{w}_m}$ can be generated from it by using the inverse, the random sample of ${W}$ can be transformed into a random sample of ${\xi }$ *via* the inverse ${{\xi }^{(1)}} = {Q}({u,}{{w}_1}), \ldots ,{{\xi }^{(m)}} = {Q}({u,}{{w}_m})$. However, in most cases, the inverse does not exist; therefore, Hannig (2009, 2013) provided the following solutions.

The structural equation, represented as $V = ({V_1},{V_2},...,{V_{{n_{(1)}}}})$, so that ${U_i} = {V_i}(\xi ,W)$ For $i = 1,2, \ldots ,{n_{(1)}}.$ Let ${W} = ({W_1}, \ldots ,{W_{{n_{(1)}}}})$ are i.i.d. samples from a Uniform (0, 1) distribution, assumes that the parameter ${{\xi }}\, \in \,\Xi \, \subseteq {{\mathbb R}^p}$ is p-dimensional. Hannig (2009) showed that the generalized fiducial distribution was continuous with density.

(26) $$\eta ({\boldsymbol\xi }) = {{J({{ u},{\boldsymbol\xi } })L({ u},{\boldsymbol\xi })} \over {\int\limits_\Xi  {J({{ u},{\boldsymbol\xi }^{\prime}})L({{ u},{\boldsymbol\xi }^{\prime}})d\xi ^{\prime}}}},$$

where $L({u},{{\xi }})$ represents the likelihood of the data, and the function $J({u},{{\xi }})$ is defined as follows:

(27) $$J({u},{{\xi }}) = \sum\limits_{\scriptstyle i = ({i_1}, \ldots ,{i_p}) \atop \scriptstyle 1 \le {i_1} < \cdots < {i_p} \le {n_{(1)}} } {\left| {\det {{\left( {{{\left( {\displaystyle{{d} \over {{du}}}{{V}^{ - 1}}({u,\xi })} \right)}^{ - 1}}\displaystyle{{d} \over {{d\xi }}}{{V}^{ - 1}}({u,\xi })} \right)}_i}} \right|} ,$$

where the sum covers all *p*-tuples of indexes $i = (1 \le {i_1} < \cdots < {i_p} \le {n_{(1)}}) \subset \{ 1, \ldots ,{n_{(1)}}\}$ and $d{{V}^{ - 1}}(u{,\xi })d{\xi }$ is the ${n_{(1)}} \times p$ and $d{{V}^{ - 1}}({u,\xi })/d{u}$ is ${n_{(1)}} \times {n_{(1)}}$ Jacobian matrices. For any ${n_{(1)}} \times p$ matrix $Y$, submatrix $({Y_1})$ is a $p \times p$ matrix containing the rows ${i_1}, \ldots ,{1_p}$ of $Y$. Hannig (2009) demonstrated that if the sample $u$ was independently and identically distributed (i.i.d.) from a distribution with a continuous cumulative distribution function (CDF) denoted as ${F_\xi }(u),$ then ${{V}^{ - 1}} = ({F_\xi }({u_1}), \ldots ,{F_\xi }({u_{{n_{(1)}}}}))$.

In the case of the BirSau distribution, it involves two parameters, denoted as $\alpha$ and $\beta$. Therefore, the generalized fiducial distribution of $\alpha$ and $\beta$ is as follows:

(28) $$q\left( {\alpha ,\beta \left| x \right.} \right)\, \propto \,L(x\left| {\alpha ,\beta } \right.)J\left( {x,(\alpha ,\beta )} \right),$$

where $L\left( {x\left| {\alpha ,\beta } \right.} \right)$ represents the joint likelihood of the observed data $x$, defined as follows:

(29) $$L\left( {x\left| {\alpha ,\beta } \right.} \right) = {\left( {\displaystyle{1 \over {2\sqrt {2\pi } \alpha \beta }}} \right)^{{n_{(1)}}}}\prod\limits_{i = 1}^{{n_{(1)}}} {\left[ {{{\left( {\displaystyle{\beta \over {{x_i}}}} \right)}^{1/2}} + {{\left( {\displaystyle{\beta \over {{x_i}}}} \right)}^{3/2}}} \right]} \exp \left[ { - \displaystyle{1 \over {2{\alpha ^2}}}\sum\limits_{i = 1}^{{n_{(1)}}} {\left( {\displaystyle{{{x_i}} \over \beta } + \displaystyle{\beta \over {{x_i}}} - 2} \right)} } \right],$$

and

(30) $$J({x},(\alpha ,\beta )) = \sum\limits_{1 \le i < j \le {n_{(1)}}} {\displaystyle{{4\left| {{x_i} - {x_j}} \right|} \over {\alpha (1 + \beta /{x_i})(1 + \beta /{x_j})}}} .$$

In fact, $J({x},(\alpha ,\beta ))$ plays a role similar to that of the prior distribution in the Bayesian framework, resembling a data-dependent prior. Equation (30) indicates that $\alpha$ and $\beta$ are independent of one another, Li & Xu (2016) showed that the prior of $\alpha$ and $\beta$ can be expressed as follows:

(31) $$\eqalign{& \pi \left( \alpha \right) \propto \displaystyle{1 \over \alpha }, \cr & \pi \left( \beta \right) \propto \sum\limits_{1 \le i < j \le {n_{(1)}}} {\displaystyle{{\left| {{x_i} - {x_j}} \right|} \over {(1 + \beta /{x_i})\left( {1 + \beta /{x_j}} \right)}}} .}$$

Note that the symbol $\propto$ means “is proportional to.”

Thus, $f\left( {\alpha ,\beta \left| x \right.} \right)$ is appropriate for the specific case of a prior with partial information given by the parameters $\alpha$ and $\beta$. The generalized fiducial samples of $\alpha$ and $\beta$, denoted as $O_\alpha ^{GFCI}$ and $O_\beta ^{GFCI}$, can be obtained from the generalized fiducial distribution like the Bayesian posterior. Gilks & Wild (1992) introduced the adaptive rejection Metropolis sampling (ARMS), originating from adaptive rejection sampling (ARS), to generate the fiducial samples $O_\alpha ^{GFCI}$ and $O_\beta ^{GFCI}$ from the generalized fiducial distribution Eq. (28). ARS, developed by Gilks & Wild (1992), utilizes log-concave target densities. To address the limitations of ARS, Gilks, Best & Tan (1995) expanded the algorithm to handle multivariate distributions and non-log-concave distributions by allowing the proposal distribution to remain below the target in specific regions and incorporating a Metropolis-Hastings step to ensure proper distribution of the accepted samples.

The *“arms”* function in the R software is *the “dlm” package, which* can be used to run the provided algorithm, which is known as ARMS easily. Be aware that the variables $O_\alpha ^{GFCI}$ and $O_\beta ^{GFCI}$ are considered as random. Thus, $\alpha$ and $\beta$ are changed to $O_\alpha ^{GFCI}$ and $O_\beta ^{GFCI}$, respectively.

#### The generalized fiducial confidence interval based on the VST method (GFCI-1)

By incorporating $O_\delta ^{VST}$ in Eq. (8), along with the generalized fiducial estimators $O_\alpha ^{GFCI}$ and $O_\beta ^{GFCI}$, the generalized fiducial estimator of $\tau$ based on the VST method, it can be expressed as:

(32) $$ O_\tau ^{GFCI.VST} = (1 - O_\delta ^{VST}){(O_\beta ^{GFCI})^2}\left( {{{\left( {O_\alpha ^{GFCI}} \right)}^2}\left( {1 + \displaystyle{5 \over 4}{{\left( {O_\alpha ^{GFCI}} \right)}^2}} \right) + O_\delta ^{VST}{{\left( {1 + \displaystyle{1 \over 2}{{\left( {O_\alpha ^{GFCI}} \right)}^2}} \right)}^2}} \right).$$

Therefore, the $100(1 - \gamma )\%$ confidence interval for $\tau$ using the VST method for GFCI is

(33) $$CI_\tau ^{GFCI.VST} = \,[O_\tau ^{GFCI.VST}(\gamma /2),O_\tau ^{GFCI.VST}(1 - \gamma /2)],$$

where $O_\tau ^{GFCI.VST}(\gamma /2)$ and $O_\tau ^{GFCI.VST}(1 - \gamma /2)$ are the $100{(\gamma /2)^{th}}$ and $100{(1 - \gamma /2)^{th}}$ percentiles of the distribution of $O_\tau ^{GFCI.VST}$, respectively.

#### The generalized fiducial confidence interval based on the Wilson score method (GFCI-2)

By employing Eq. (11) along with $O_\delta ^W$, as well as the generalized fiducial estimators $O_\alpha ^{GFCI}$ and $O_\beta ^{GFCI}$, the generalized fiducial estimator of $\tau$ based on the Wilson score method can be formulated as follows:

(34) $$ O_\tau ^{GFCI.W} = (1 - O_\delta ^W){(O_\beta ^{GFCI})^2}\left( {{{\left( {O_\alpha ^{GFCI}} \right)}^2}\left( {1 + \displaystyle{5 \over 4}{{\left( {O_\alpha ^{GFCI}} \right)}^2}} \right) + O_\delta ^W{{\left( {1 + \displaystyle{1 \over 2}{{\left( {O_\alpha ^{GFCI}} \right)}^2}} \right)}^2}} \right).$$

Therefore, the $100(1 - \gamma )\%$ confidence interval for $\tau$ using the Wilson score method for GFCI is

(35) $$CI_\tau ^{GFCI.W} = \,[O_\tau ^{GFCI.W}(\gamma /2),O_\tau ^{GFCI.W}(1 - \gamma /2)],$$

where $O_\tau ^{GFCI.W}(\gamma /2)$ and $O_\tau ^{GFCI.W}(1 - \gamma /2)$ are the $100{(\gamma /2)^{th}}$ and $100{(1 - \gamma /2)^{th}}$ percentiles of the distribution of $O_\tau ^{GFCI.W}$, respectively.

#### The generalized fiducial confidence interval based on the Hannig method (GFCI-3)

By integrating $O_\delta ^H$ into Eq. (14), alongside the generalized fiducial estimators $O_\alpha ^{GFCI}$ and $O_\beta ^{GFCI}$, the generalized fiducial estimator of $\tau$ utilizing the Hannig method can be expressed as follows:

(36) $$ O_\tau ^{GFCI.H} = (1 - O_\delta ^H){(O_\beta ^{GFCI})^2}\left( {{{\left( {O_\alpha ^{GFCI}} \right)}^2}\left( {1 + \displaystyle{5 \over 4}{{\left( {O_\alpha ^{GFCI}} \right)}^2}} \right) + O_\delta ^H{{\left( {1 + \displaystyle{1 \over 2}{{\left( {O_\alpha ^{GFCI}} \right)}^2}} \right)}^2}} \right).$$

Therefore, the $100(1 - \gamma )\%$ confidence interval for $\tau$ using the Hannig method for GFCI is

(37) $$CI_\tau ^{GFCI.H} = \,[O_\tau ^{GFCI.H}(\gamma /2),O_\tau ^{GFCI.H}(1 - \gamma /2)],$$

where $O_\tau ^{GFCI.H}(\gamma /2)$ and $O_\tau ^{GFCI.H}(1 - \gamma /2)$ are the $100{(\gamma /2)^{th}}$ and $100{(1 - \gamma /2)^{th}}$ percentiles of the distribution of $O_\tau ^{GFCI.H}$, respectively.

**Algorithm 3:** GFCI

1. Define the parameters $\alpha$, $\beta$, and $\delta$.
2. Define $n$, ${n_{\left( 0 \right)}}$, and ${n_{\left( 1 \right)}}$, where ${n_{\left( 0 \right)}}$ is determined as a realization from ${n_{(0)}} \sim bi(n,\delta )$, and ${n_{\left( 1 \right)}}$
is calculated as ${n_{\left( 1 \right)}} = n - {n_{\left( 0 \right)}}$.
3. Simulate the datasets using the delta-BirSau distribution with parameters $\alpha$, $\beta$, and $\delta$, where
$X\sim{\rm delta - BirSau}(\alpha ,\beta ,\delta )$.
4. Consider datasets containing values greater than zero.
5. Generating samples of $\alpha$ and $\beta$ by using the *arms* function in the *dlm* package.
6. Burn-in $T$ samples, keeping the remaining $K - T$ samples.
7. Thin the samples by applying sampling lag $L > 1$ and the final number of iterations kept is
$K' = (K - T)/L$.
8. Since the generated sample is not independent, tinning the sample will help to lower the
autocorrelation.
9. Calculate the fiducial estimates of $\alpha$ and $\beta$ denote as $O_\alpha ^{GFCI}$ and $O_\beta ^{GFCI}$.

### Normal approximation: NA

In the normal approximation method, we utilize the Delta method to achieve a normal distribution as the limiting distribution of an estimator $\tau$. This method, which is widely recognized, can be briefly described as follows:

Let $g({u_1},{u_2},{u_3})$ represent a differentiable scalar function of three parameters. We have an estimator, $G = g({U_1},{U_2},{U_3})$, which depends on three parameters: ${U_1},{U_2}$ and ${U_3}$. Utilizing the Delta method, we can determine the asymptotic distribution of $G$, which involves providing a stochastic representation of $G$. We apply the Delta method to establish the asymptotic normality of $G$ as the sample sizes approach infinity and to compute the asymptotic mean and variance of $\tau$. In the Delta method, the function $g$ is used to expand into the Taylor series at the point ${\theta _1},{\theta _2},{\theta _3}$ of ${U_1},{U_2},{U_3}$ as follows

(38) $$\eqalign{g({U_1},{U_2},{U_3})& \approx g({\theta _1},{\theta _2},{\theta _3}) + \displaystyle{{\partial g({\theta _1},{\theta _2},{\theta _3})} \over {\partial {u_1}}}\left( {{U_1} - {\theta _1}} \right) + \displaystyle{{\partial g({\theta _1},{\theta _2},{\theta _3})} \over {\partial {u_2}}}\left( {{U_2} - {\theta _2}} \right)\\& + \displaystyle{{\partial g({\theta _1},{\theta _2},{\theta _3})} \over {\partial {u_3}}}\left( {{U_3} - {\theta _3}} \right).}$$

The basic statistic was assumed here as ${U_1} = \hat \alpha$, ${U_2} = \hat \beta$, and ${U_3} = \hat \delta$ where $\hat \alpha ,\hat \beta$ are the MMEs of $\alpha ,$ and $\beta$, respectively and $\hat \delta$ is the MLEs of $\delta$. Then, $\hat \tau = g(\hat \alpha ,\hat \beta ,\hat \delta )$, where the function $g(\alpha ,\beta ,\delta ) = (1 - \delta )({\beta ^2})[{\alpha ^2}(1 + 1.25{\alpha ^2}) + \delta {(1 + 0.5{\alpha ^2})^2}]$. The partial derivatives are as follows

$$\displaystyle{{\partial g(\alpha ,\beta ,\delta )} \over {\partial \alpha }} = - {\beta ^2}\left( {\delta - 1} \right)\left( {2\alpha \left( {\displaystyle{{5{\alpha ^2}} \over 4} + 1} \right) + \displaystyle{{5{\alpha ^3}} \over 2} + 2\alpha \delta \left( {\displaystyle{{{\alpha ^2}} \over 2} + 1} \right)} \right).$$

$$\displaystyle{{\partial g(\alpha ,\beta ,\delta )} \over {\partial \beta }} = - 2\beta \left[ {{\alpha ^2}\left( {\displaystyle{{5{\alpha ^2}} \over 4} + 1} \right) + \delta {{\left( {\displaystyle{{{\alpha ^2}} \over 2} + 1} \right)}^2}} \right]\left( {\delta - 1} \right).$$

$$\displaystyle{{\partial g(\alpha ,\beta ,\delta )} \over {\partial \delta }} = - 2{\beta ^2}\left[ {{\alpha ^2}\left( {\left( {\displaystyle{{5{\alpha ^2}} \over 4} + 1} \right) + \delta {{\left( {\displaystyle{{{\alpha ^2}} \over 2} + 1} \right)}^2}} \right)} \right] - {\beta ^2}{\left( {\displaystyle{{{\alpha ^2}} \over 2} + 1} \right)^2}(\delta - 1).$$

Therefore

(39) $$\eqalign{  \hat \tau \approx \; & g\left( {\alpha ,\beta ,\delta } \right) + \displaystyle{{\partial g\left( {\alpha ,\beta ,\delta } \right)} \over {\partial \alpha }}\left( {\hat \alpha - \alpha } \right) + \displaystyle{{\partial g\left( {\alpha ,\beta ,\delta } \right)} \over {\partial \beta }}\left( {\hat \beta - \beta } \right) + \displaystyle{{\partial g\left( {\alpha ,\beta ,\delta } \right)} \over {\partial \delta }}\left( {\hat \delta - \delta } \right) \cr & = g\left( {\alpha ,\beta ,\delta } \right) + \left( {\left( {1 - \delta } \right)\left( {{\beta ^2}} \right)\left( {2\alpha + 5{\alpha ^3} + 2\alpha \delta + {\alpha ^3}\delta } \right)} \right)\left( {\hat \alpha - \alpha } \right) \cr &  + \left( {2\beta \left( {1 - \delta } \right)\left( {{\alpha ^2}\left( {1 + 1.25{\alpha ^2}} \right) + \delta {{\left( {1 + 0.5{\alpha ^2}} \right)}^2}} \right)} \right)\left( {\hat \beta - \beta } \right) \cr &   - \left( {{\beta ^2}\left( {{\alpha ^2}\left( {1 + 1.25{\alpha ^2}} \right) - {{(1 + 0.5{\alpha ^2})}^2} + 2\delta {{\left( {1 + 0.5{\alpha ^2}} \right)}^2}} \right)} \right)(\hat \delta - \delta ).}$$

Taking the expectation and variance in Eq. (39), the approximate mean and variance for the $\hat \tau$ is given by

(40) $$E\left( {\hat \tau } \right) \approx \tau,$$

(41) $$\eqalign{ & Var(\hat \tau ) \approx Var[g\left( {\alpha ,\beta ,\delta } \right) + \left( {\left( {1 - \delta } \right)\left( {{\beta ^2}} \right)\left( {2\alpha + 5{\alpha ^3} + 2\alpha \delta + {\alpha ^3}\delta } \right)} \right)\left( {\hat \alpha - \alpha } \right) \cr & \,\,\,\,\,\,\,\,\,\,\,\,\,\,\,\,\,\,\,\,\, + \left( {2\beta \left( {1 - \delta } \right)\left( {{\alpha ^2}\left( {1 + 1.25{\alpha ^2}} \right) + \delta {{\left( {1 + 0.5{\alpha ^2}} \right)}^2}} \right)} \right)\left( {\hat \beta - \beta } \right) \cr & \,\,\,\,\,\,\,\,\,\,\,\,\,\,\,\,\,\,\,\,\, - \left( {{\beta ^2}\left( {{\alpha ^2}\left( {1 + 1.25{\alpha ^2}} \right) - {{(1 + 0.5{\alpha ^2})}^2} + 2\delta {{\left( {1 + 0.5{\alpha ^2}} \right)}^2}} \right)} \right)(\hat \delta - \delta ) \cr & \,\,\,\,\,\,\,\,\,\,\,\,\,\,\,\,\,\,\,\,\, = {\left( {\left( {1 - \delta } \right)\left( {{\beta ^2}} \right)\left( {2\alpha + 5{\alpha ^3} + 2\alpha \delta + {\alpha ^3}\delta } \right)} \right)^2}({{0.5{\alpha ^2}} \mathord{\left/ {\vphantom {{0.5{\alpha ^2}} {{n_{(1)}}}}} \right. } {{n_{(1)}}}}) \cr & \,\,\,\,\,\,\,\,\,\,\,\,\,\,\,\,\,\,\,\,\, + {\left( {2\beta \left( {1 - \delta } \right)\left( {{\alpha ^2}\left( {1 + 1.25{\alpha ^2}} \right) + \delta {{\left( {1 + 0.5{\alpha ^2}} \right)}^2}} \right)} \right)^2}\left( {\displaystyle{{{{\left( {\alpha \beta } \right)}^2}\left( {1 + 0.75{\alpha ^2}} \right)} \over {{n_{(1)}}{{(1 + 0.5{{\hat \alpha }^2})}^2}}}} \right) \cr & \,\,\,\,\,\,\,\,\,\,\,\,\,\,\,\,\,\,\,\,\, + {\left( {{\beta ^2}\left( {{\alpha ^2}\left( {1 + 1.25{\alpha ^2}} \right) - {{(1 + 0.5{\alpha ^2})}^2} + 2\delta {{\left( {1 + 0.5{\alpha ^2}} \right)}^2}} \right)} \right)^2}\left( {\displaystyle{{\delta (1 - \delta )} \over n}} \right).}$$

Therefore, as ${n_i} \rightarrow \infty$, $\hat \tau$ is approximately normal $N(E(\hat \tau ),Var\left( {\hat \tau } \right))$. Obviously, value $\alpha ,\beta ,$ and $\delta$ are unknown when we estimate the parameter function $\tau$ have only samples in our hands. Then, we use the plug-in estimators of $E\left( {\hat \tau } \right)$ and $Var(\hat \tau )$ as follows

(42) $$\hat E(\hat \tau ) = (1 - \hat \delta )\left( {{{\hat \beta }^2}} \right)\left( {{{\hat \alpha }^2}\left( {1 + \displaystyle{5 \over 4}{{\hat \alpha }^2}} \right) + \hat \delta {{\left( {1 + \displaystyle{1 \over 2}{{\hat \alpha }^2}} \right)}^2}} \right),$$

(43) $$\eqalign{ \hat V{ar} (\hat \tau ) =& \; {\left( {\left( {1 - \hat \delta } \right)\left( {{{\hat \beta }^2}} \right)\left( {2\hat \alpha + 5{{\hat \alpha }^3} + 2\hat \alpha \hat \delta + {{\hat \alpha }^3}\hat \delta } \right)} \right)^2}({{0.5{{\hat \alpha }^2}} \mathord{\left/ { {{0.5{{\hat \alpha }^2}} {{n_{(1)}}}}} \right. } }) \cr &  + {\left( {2\hat \beta \left( {1 - \hat \delta } \right)\left( {{{\hat \alpha }^2}\left( {1 + 1.25{{\hat \alpha }^2}} \right) + \hat \delta {{\left( {1 + 0.5{{\hat \alpha }^2}} \right)}^2}} \right)} \right)^2}\left( {\displaystyle{{{{\left( {\hat \alpha \hat \beta } \right)}^2}\left( {1 + 0.75{{\hat \alpha }^2}} \right)} \over {{n_{(1)}}{{(1 + 0.5{{\hat \alpha }^2})}^2}}}} \right) \cr & + {\left( {{{\hat \beta }^2}\left( {{{\hat \alpha }^2}\left( {1 + 1.25{{\hat \alpha }^2}} \right) - {{(1 + 0.5{{\hat \alpha }^2})}^2} + 2\hat \delta {{\left( {1 + 0.5{{\hat \alpha }^2}} \right)}^2}} \right)} \right)^2}\left( {\displaystyle{{\hat \delta (1 - \hat \delta )} \over n}} \right).}$$

**Algorithm 4:** NA

1. Define the parameters $\alpha$, $\beta$, and $\delta$.
2. Define $n$, ${n_{\left( 0 \right)}}$, and ${n_{\left( 1 \right)}}$, where ${n_{\left( 0 \right)}}$ is determined as a realization from ${n_{(0)}} \sim bi(n,\delta )$, and ${n_{\left( 1 \right)}}$
is calculated as ${n_{\left( 1 \right)}} = n - {n_{\left( 0 \right)}}$.
3. Simulate the datasets using the delta-BirSau distribution with parameters $\alpha$, $\beta$, and $\delta$, where
$X\sim{\rm delta - BirSau}(\alpha ,\beta ,\delta )$.
4. Calculate the method of MME for the parameters $\alpha$ and $\beta$, which are denoted as $\hat \alpha$ and $\hat \beta$,
respectively. Then, compute the MLE for the parameter $\delta$, denoted as $\hat \delta$.
5. Then, use $\hat \alpha$, $\hat \beta$, and $\hat \delta$ to compute $\tau$ and obtain $\hat \tau$.
6. Compute the 95% NA confidence interval for $\tau$ by using Eq. (45).

The asymptotically standard normal distribution is

(44) $$Z = \displaystyle{{\hat \tau - \tau } \over {\sqrt {\hat V {ar} (\hat \tau )} }} \sim N(0,1).$$

Therefore, the $100(1 - \gamma )\%$ confidence interval for $\tau$ using normal approximation is

(45) $$CI_\tau ^{NA} = \,\hat \tau \pm {z_{1 - {\gamma \mathord{\left/ { 2} \right. } }}}\sqrt {\hat V {ar} (\hat \tau )} ,$$

where ${Z_{1 - {\gamma \mathord{\left/ {\vphantom {\gamma 2}} \right. } 2}}}$ is the ${\left( {1 - {\gamma \mathord{\left/ {\vphantom {\gamma 2}} \right. } 2}} \right)^{th}}$ percentile of the standard normal distribution.

## Results

The study utilized the R statistical software to conduct a simulation, explicitly employing a Monte Carlo simulation to evaluate the performance of confidence intervals for the variance of the delta-BirSau distribution. The investigation compared the effectiveness of ten methods (GCI-1,2,3, BCI-1,2,3, GFCI-1,2,3, and NA) by examining their CPs and AWs. First, the evaluation focused on the confidence intervals based on their CPs. With a nominal confidence level of 0.95, the selection criteria were set to include intervals that provided CPs close to or greater than the nominal confidence level of 0.95. Following this, the AWs of these chosen confidence intervals were considered to identify the interval with the shortest width as the optimal confidence interval. This comprehensive approach ensures a thorough assessment of the performance of the different methods under consideration. The data were generated for $X\sim{\rm delta} - {\rm BirSau}(\alpha ,\beta ,\delta )$ with sample sizes $n = 15,20,30,50,70,$ or 100 and probabilities of zeros $\delta = 0.1,0.3,$ or 0.5 (except in the case of $n = 15$, only $\delta$ values of 0.1 and 0.3 will be considered), where we set $\alpha = 0.25,0.50,0.75,$ or 1. Additionally, we set $\beta = 1$ or 2 for all cases. In this study, we set various parameters because different values of $\alpha$ results in different slopes of the function, and various values of $\beta$ affect the spread of the distribution, as shown in Fig. 1. For the parameter $\alpha$, it defines the shape of the data distribution. Specifically, if $\alpha$ is low, the data distribution will be symmetric or close to a normal distribution. As $\alpha$ increases, the distribution becomes more right-skewed. The parameter $\beta$ determines the scale or spread of the data distribution. A higher $\beta$ value indicates a wider data distribution, resulting in a broader range of data. The parameter $\delta$ indicates the proportion of zero values. If $\delta$ is high, it means that there are many zero values in the data. The number of generated random samples was fixed at 2,000 replications with *I* = 5,000 pivotal quantities for the GCI, $B = 500$ for the BCI, and *K* = 3,000, *T* = 1,000 with a thinning sample $(L)$ set to 2 for the GFCI.

**Figure 2:**
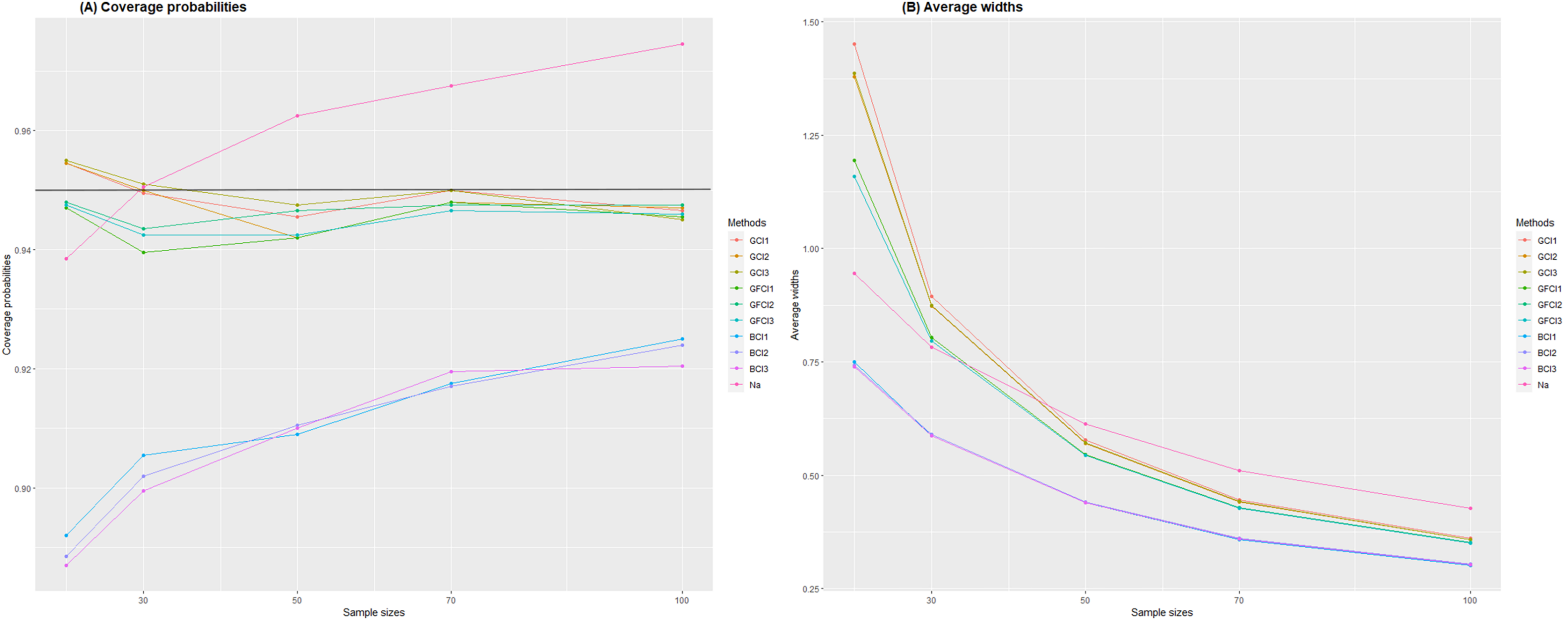
Comparison of the (A) coverage probabilities and (B) average widths for estimation of the 95% confidence interval for the variance of delta-BirSau distribution for various sample sizes. (A) Coverage probabilities and (B) average widths.

We present the CPs and AWs of nominal 95% two-sided confidence intervals for the variance of the delta-BirSau distribution in Table 1. The summary of CPs and AWs is further depicted in Fig. 2. The simulation results in Table 1 indicate that the CPs of the GCI-1,2,3, and GFCI-1,2,3 confidence intervals were consistently greater than or close to the nominal confidence level of 0.95 in all situations studied, except in the case of small sample sizes $n = 15,20,$ and 30, where the CPs of GCI-1,3 were lower than the nominal confidence level. As the sample sizes increased, the CPs of the GCI-1 and 3 performed better. For the NA method, it was observed that for medium ( $n$ = 50, 70) and large ( $n$ = 100) sample sizes with $\alpha = 0.25,0.5$, the NA method yielded a CP value greater than the nominal confidence level of 0.95. Next, we examine the values of AWs. It is observed that BCI-1,2,3 yields the shortest AW values, while the NA method produces the longest AW values. However, since both methods result in CP values below the nominal confidence level of 0.95, we choose not to consider them. Hence, we exclusively considered methods with CP values exceeding the nominal confidence level of 0.95 for our AW comparison. In the context of small sample sizes ( $n$ = 15, 20, 30), the GFCI-2 method consistently yielded the shortest AW values, except in instances where $\alpha$ = 0.25, where the GCI-1 method outperformed. Transitioning to medium-sized samples ( $n$ = 50, 70), the GFCI-3 method showcased the shortest AW values. Meanwhile, in larger sample sizes ( $n$ = 100), the GFCI-1 method exhibited the shortest AW values. Upon comprehensive analysis, it becomes apparent that the GCI-1,2,3 and GFCI-1,2,3 methods show similar AW values with minimal differences.

**Table 1:** The coverage probabilities and (average widths) of nominal 95% two-sided confidence interval for variance of delta-BirSau distribution.

$ n$	$\boldsymbol \delta$	$\boldsymbol \beta$	$\boldsymbol \alpha$	Coverage probability (Average width)									
				GCI-1	GCI-2	GCI-3	GFCI-1	GFCI-2	GFCI-3	BCI-1	BCI-2	BCI-3	NA
15	0.1	1	0.25	0.9425	0.6305	0.6640	0.9345	**0.9725**	0.9080	0.8915	**0.9705**	0.8865	0.8395
				(0.2131)	(0.2150)	(0.2168)	(0.1829)	*(0.2731)*	(0.2411)	(0.1333)	(0.2368)	(0.1985)	(0.3364)
			0.5	**0.9525**	0.9335	0.9395	**0.9450**	**0.9480**	0.9400	0.8990	0.9070	0.8995	0.8745
				(1.5890)	(1.5590)	(1.5610)	(1.2970)	*(1.2930)*	(1.2920)	(0.8210)	(0.8382)	(0.8299)	(0.8562)
			0.75	**0.9460**	0.9445	**0.9460**	0.9395	0.9395	0.9390	0.8885	0.8865	0.8875	0.8125
				(7.6860)	(7.3980)	*(7.4200)*	(5.7990)	(5.6670)	(5.6690)	(3.1770)	(3.1350)	(3.1300)	(2.6630)
			1	**0.9465**	**0.9455**	**0.9460**	0.9320	0.9315	0.9315	0.8735	0.8740	0.8745	0.7695
				(26.8200)	(25.9600)	*(25.8100)*	(19.4300)	(18.9700)	(18.9700)	(8.9470)	(8.8220)	(8.7880)	(6.9200)
		2	0.25	**0.9495**	0.6255	0.6600	0.9325	**0.9630**	0.9315	0.8835	**0.9595**	0.8865	0.8460
				(0.8396)	(0.8491)	(0.8551)	(0.7185)	(1.0834)	(0.9555)	(0.5242)	*(0.9422)*	(0.7885)	(1.3450)
			0.5	**0.9520**	0.9390	0.9445	0.9430	**0.9505**	0.9445	0.9015	0.9140	0.9070	0.8715
				(6.2750)	(6.1460)	(6.1630)	(5.0930)	*(5.1160)*	(5.0960)	(3.2640)	(3.3320)	(3.2980)	(3.4080)
			0.75	**0.9480**	**0.9495**	**0.9475**	0.9430	0.9440	0.9445	0.8930	0.8925	0.8920	0.8145
				(29.1200)	*(28.1800)*	(28.2600)	(22.4100)	(21.9500)	(21.9500)	(12.2500)	(12.1100)	(12.0900)	(10.3700)
			1	**0.9560**	**0.9545**	**0.9560**	0.9410	**0.9455**	0.9415	0.8935	0.8930	0.8955	0.7940
				(108.3000)	(103.9000)	(103.8000)	(77.9700)	*(75.9300)*	(75.8900)	(36.0500)	(35.5000)	(35.3600)	(27.8100)
	0.3	1	0.25	0.9445	0.8735	0.8750	0.9335	**0.9480**	0.9215	0.8815	0.9160	0.8825	**0.9815**
				(0.3373)	(0.3188)	(0.3207)	(0.2758)	*(0.3128)*	(0.2971)	(0.1876)	(0.2357)	(0.2169)	(0.5416)
			0.5	**0.9485**	**0.9450**	**0.9465**	0.9310	0.9310	0.9280	0.8810	0.8720	0.8730	0.9210
				(2.5580)	(2.3610)	*(2.3310)*	(1.8060)	(1.7060)	(1.7090)	(0.9276)	(0.9113)	(0.9065)	(1.0970)
			0.75	**0.9535**	**0.9530**	**0.9510**	0.9420	0.9375	0.9370	0.8780	0.8695	0.8685	0.8200
				(17.7700)	*(13.1400)*	(14.2300)	(21.3700)	(16.1700)	(15.1300)	(3.4000)	(3.2400)	(3.2250)	(2.8430)
			1	**0.9475**	**0.9445**	**0.9450**	0.9310	**0.9452**	0.9285	0.8575	0.8520	0.8535	0.767
				(55.5100)	(48.8700)	(49.8800)	(32.5300)	*(29.5700)*	(29.7000)	(9.2450)	(9.0170)	(8.9650)	(7.0050)
		2	0.25	**0.9535**	0.8720	0.8865	0.9395	**0.9500**	0.9335	0.8975	0.9150	0.8820	**0.9780**
				(1.3340)	(1.2520)	(1.2720)	(1.0930)	*(1.2380)*	(1.1750)	(0.7471)	(0.9368)	(0.8620)	(2.1350)
			0.5	0.9430	0.9400	0.9420	0.9285	0.9300	0.9310	0.8685	0.8495	0.8525	0.9035
				(9.7930)	(8.9720)	(9.0170)	(6.9350)	(6.5560)	(6.5650)	(3.6270)	(3.5750)	(3.5560)	(4.2970)
			0.75	0.9405	0.9445	0.9445	0.9320	0.9355	0.9360	0.8745	0.8740	0.8740	0.8200
				(62.6000)	(54.2200)	(52.4500)	(38.6500)	(34.9000)	(34.1100)	(13.5900)	(13.2400)	(13.2000)	(11.6000)
			1	0.9440	0.9390	0.9430	0.9435	0.9405	0.9415	0.8710	0.8665	0.8675	0.7840
				(245.4000)	(213.3000)	(218.7000)	(128.5000)	(118.7000)	(119.5000)	(38.4000)	(37.7500)	(37.5700)	(28.9200)
20	0.1	1	0.25	**0.9475**	0.6185	0.6455	0.9395	**0.9590**	0.9000	0.9055	**0.9600**	0.8820	0.8910
				*(0.1630)*	(0.1653)	(0.1660)	(0.1464)	(0.2311)	(0.1996)	(0.1150)	(0.2094)	(0.1732)	(0.2953)
			0.5	**0.9510**	0.9360	0.9400	**0.9450**	**0.9500**	0.9420	0.9050	0.9195	0.9105	0.8990
				(1.0753)	(1.0634)	(1.0653)	*(0.9362)*	(0.9493)	(0.9443)	(0.6748)	(0.6941)	(0.6862)	(0.7377)
			0.75	**0.9535**	**0.9545**	**0.9570**	0.9430	**0.9455**	**0.9455**	0.9175	0.9195	0.9200	0.8515
				(4.7134)	(4.6285)	(4.6285)	(4.0137)	*(3.9616)*	(3.9629)	(2.5903)	(2.5718)	(2.5720)	(2.2680)
			1	**0.9520**	**0.9525**	**0.9510**	0.9410	0.9410	0.9415	0.9115	0.9110	0.9125	0.8175
				(15.1140)	*(14.7576)*	(14.7986)	(12.2197)	(12.0233)	(12.0221)	(7.1196)	(7.0550)	(7.0442)	(5.8910)
		2	0.25	**0.9530**	0.6190	0.6500	0.9435	**0.9640**	0.8900	0.9060	**0.9620**	0.8765	0.8785
				*(0.6468)*	(0.6546)	(0.6579)	(0.5816)	(0.9216)	(0.7931)	(0.4560)	(0.8347)	(0.6888)	(1.1920)
			0.5	**0.9555**	0.9360	0.9385	0.9435	**0.9530**	**0.9515**	0.8955	0.9130	0.9075	0.9065
				(4.3649)	(4.3130)	(4.3219)	(3.7909)	*(3.8436)*	(3.8228)	(2.7325)	(2.8070)	(2.7780)	(2.9910)
			0.75	**0.9580**	**0.9590**	**0.9585**	**0.9570**	**0.9575**	**0.9575**	0.9225	0.9230	0.9240	0.8435
				(18.3736)	(17.9767)	(18.0348)	(15.5455)	*(15.3393)*	(15.3412)	(10.0565)	(9.9822)	(9.9770)	(9.0900)
			1	**0.9455**	0.9435	**0.9455**	0.9355	0.9350	0.9350	0.9045	0.9020	0.9030	0.8190
				(61.7004)	(60.2375)	*(60.2959)*	(50.6327)	(50.6233)	(49.8119)	(29.1641)	(28.8883)	(28.8554)	(23.9800)
	0.3	1	0.25	0.9445	0.8635	0.8770	0.9385	**0.9545**	0.9305	0.9015	0.9285	0.8980	**0.9875**
				(0.2419)	(0.2318)	(0.2345)	(0.2139)	*(0.2514)*	(0.2357)	(0.1607)	(0.2047)	(0.1872)	(0.4694)
			0.5	**0.9545**	**0.9545**	**0.9550**	**0.9470**	**0.9480**	**0.9475**	0.8920	0.8885	0.8870	0.9385
				(1.4508)	(1.3783)	(1.3869)	(1.1948)	(1.1589)	*(1.1588)*	(0.7505)	(0.7430)	(0.7399)	(0.9446)
			0.75	**0.9520**	**0.9535**	**0.9500**	**0.9455**	**0.9465**	**0.9460**	0.9000	0.8945	0.8945	0.8435
				(6.4893)	(6.1149)	(6.1939)	(4.9919)	*(4.7801)*	(4.7911)	(2.6496)	(2.6114)	(2.6031)	(2.4770)
			1	**0.9485**	**0.9500**	**0.9515**	0.9415	0.9380	0.9375	0.8935	0.8890	0.8875	0.8105
				(23.2626)	*(21.5508)*	(21.8330)	(17.4127)	(16.5339)	(16.5161)	(7.6380)	(7.5258)	(7.4775)	(6.3200)
		2	0.25	**0.9480**	0.8495	0.8720	0.9400	**0.9535**	0.9310	0.8915	0.9240	0.8920	**0.9855**
				*(0.9659)*	(0.9248)	(0.9346)	(0.8512)	(1.0032)	(0.9395)	(0.6407)	(0.8202)	(0.7488)	(1.8690)
			0.5	0.9425	0.9420	0.9420	0.9295	0.9315	0.9285	0.8730	0.8610	0.8650	0.9335
				(5.5713)	(5.3281)	(5.3754)	(4.6136)	(4.5016)	(4.5047)	(2.9109)	(2.9036)	(2.8912)	(3.7450)
			0.75	**0.9585**	**0.9605**	**0.9595**	**0.9470**	**0.9485**	**0.9480**	0.8930	0.8835	0.8865	0.8445
				(25.4888)	(24.1294)	(24.0169)	(19.8831)	*(19.1142)*	(19.1330)	(10.6235)	(10.4913)	(10.4599)	(9.7660)
			1	**0.9535**	**0.9495**	**0.9470**	0.9400	0.9445	0.9425	0.8960	0.8955	0.8970	0.8230
				(93.0116)	*(87.9056)*	(88.4969)	(65.3842)	(62.9130)	(63.1393)	(29.6448)	(29.4250)	(29.2789)	(25.5800)
20	0.5	1	0.25	**0.9505**	0.9215	0.9370	0.9315	0.9350	0.9330	0.8860	0.8745	0.8650	**0.9960**
				*(0.4109)*	(0.3324)	(0.3379)	(0.2875)	(0.2893)	(0.2792)	(0.1935)	(0.2106)	(0.1989)	(0.5204)
			0.5	**0.9460**	0.9440	**0.9460**	0.9360	0.9395	0.9330	0.8675	0.8615	0.8600	0.9230
				(2.6429)	(2.2317)	(2.2363)	(1.6585)	(1.5446)	(1.5424)	(0.8065)	(0.8022)	(0.7912)	(0.9943)
			0.75	0.9445	0.9365	0.9400	0.9275	0.9320	0.9285	0.8615	0.8645	0.8645	0.8285
				(39.4495)	(20.9059)	(25.0844)	(8.8225)	(7.6001)	(7.5236)	(2.7939)	(2.7492)	(2.7176)	(2.4840)
			1	0.9420	**0.9475**	**0.9455**	0.9315	0.9360	0.9370	0.8570	0.8545	0.8550	0.7800
				(148.9182)	(76.8764)	*(72.3319)*	(31.6468)	(26.0205)	(25.9969)	(7.3252)	(7.1907)	(7.1117)	(5.8910)
		2	0.25	**0.9465**	0.9240	0.9320	0.9340	0.9400	0.9330	0.8835	0.8730	0.8650	**0.9960**
				*(1.4022)*	(1.2657)	(1.2867)	(1.1223)	(1.1455)	(1.1060)	(0.7694)	(0.8387)	(0.7925)	(2.0860)
			0.5	**0.9505**	0.9425	**0.9485**	0.9365	0.9400	0.9370	0.8680	0.8580	0.8565	0.9165
				(10.4660)	(9.0494)	*(8.8563)*	(6.5251)	(6.0567)	(6.0608)	(3.1751)	(3.1601)	(3.1161)	(4.0190)
			0.75	**0.9455**	0.9425	0.9370	0.9300	0.9300	0.9285	0.8720	0.8685	0.8655	0.8300
				*(325.1737)*	(120.8820)	(163.2695)	(37.1266)	(31.3527)	(31.3854)	(11.6006)	(11.4101)	(11.2944)	(10.1700)
			1	0.9410	0.9380	0.9365	0.9245	0.9205	0.9190	0.8590	0.8560	0.8535	0.7920
				(663.3882)	(463.2263)	(436.7391)	(263.2367)	(181.2472)	(174.9051)	(33.4599)	(31.9553)	(31.5560)	(23.4500)
30	0.1	1	0.25	**0.9475**	0.5735	0.6250	0.9445	**0.9580**	0.8760	0.9040	**0.9555**	0.8645	0.9335
				*(0.1171)*	(0.1187)	(0.1190)	(0.1095)	(0.1869)	(0.1566)	(0.0919)	(0.1758)	(0.1427)	(0.2493)
			0.5	0.9395	0.9315	0.9355	0.9345	0.9435	0.9375	0.9055	0.9230	0.9170	0.9140
				(0.7302)	(0.7260)	(0.7273)	(0.6733)	(0.6882)	(0.6821)	(0.5366)	(0.5549)	(0.5477)	(0.6113)
			0.75	**0.9490**	**0.9495**	**0.9485**	**0.9455**	**0.9460**	0.9445	0.9215	0.9240	0.9235	0.8705
				(2.8413)	(2.8086)	(2.8133)	(2.5716)	*(2.5556)*	(2.5569)	(1.9412)	(1.9384)	(1.9364)	(1.8350)
			1	**0.9470**	0.9445	**0.9450**	0.9370	0.9390	0.9380	0.9100	0.9100	0.9095	0.8545
				(8.7465)	(8.6015)	*(8.6243)*	(7.7365)	(7.6617)	(7.6639)	(5.4421)	(5.4127)	(5.4105)	(4.9850)
		2	0.25	**5.4105**	0.5960	0.6360	0.9390	**0.9525**	0.8885	0.9110	**0.9475**	0.8750	0.9340
				*(0.4712)*	(0.4775)	(0.4802)	(0.4410)	(0.7500)	(0.6299)	(0.3708)	(0.7050)	(0.5738)	(1.0000)
			0.5	**0.9475**	0.9360	0.9350	0.9425	**0.9460**	0.9410	0.9220	0.9290	0.9220	0.9130
				(2.9348)	(2.9164)	(2.9198)	(2.7036)	*(0.9290)*	(2.7380)	(2.1537)	(2.2236)	(2.1944)	(2.4440)
			0.75	**0.9475**	**0.9485**	**0.9500**	0.9410	0.9430	0.9415	0.9145	0.9155	0.9165	0.8655
				(11.2267)	*(11.1140)*	(11.1343)	(10.1616)	(10.1062)	(10.1106)	(7.6582)	(7.6546)	(7.6546)	(7.3050)
			1	**0.9500**	**0.9525**	**0.9505**	**0.9475**	**0.9475**	**0.9480**	0.9125	0.9080	0.9085	0.8465
				(34.5694)	(34.0292)	(33.9926)	(30.5594)	*(30.2152)*	(30.2306)	(21.4996)	(21.3644)	(21.3427)	(19.3600)
	0.3	1	0.25	**0.9475**	0.8445	0.8560	0.9445	0.9440	0.9260	0.9180	0.9325	0.9015	**0.9935**
				*(0.1732)*	(0.1686)	(0.1698)	(0.1617)	(0.1956)	(0.1810)	(0.1320)	(0.1697)	(0.1541)	(0.3874)
			0.5	**0.9495**	**0.9500**	**0.9510**	0.9395	0.9435	0.9425	0.9055	0.9020	0.8995	**0.9505**
				(0.8944)	*(0.8727)*	(0.8743)	(0.8040)	(0.7963)	(0.7958)	(0.5877)	(0.5895)	(0.5875)	(0.7825)
			0.75	0.9400	0.9425	0.9420	0.9365	0.9390	0.9385	0.8975	0.8955	0.8990	0.8910
				(3.4177)	(3.3232)	(3.3375)	(3.0016)	(2.9414)	(2.9423)	(1.9991)	(1.9863)	(1.9812)	(0.8910)
			1	0.9430	0.9435	0.9440	0.9350	0.9385	0.9390	0.9020	0.8955	0.8965	0.8610
				(10.9198)	(10.5253)	(10.5016)	(9.2306)	(8.9835)	(8.9791)	(5.5418)	(5.4824)	(5.4608)	(4.9550)
		2	0.25	**0.9475**	0.8395	0.8690	0.9390	**0.9460**	0.9305	0.9070	0.9180	0.8915	**0.9900**
				*(0.6806)*	(0.6608)	(0.6665)	(0.6356)	(0.7755)	(0.7166)	(0.5185)	(0.6741)	(0.6103)	(1.5430)
			0.5	**0.9500**	**0.9465**	**0.9495**	0.9430	**0.9470**	**0.9450**	0.8995	0.8935	0.8915	**0.9555**
				(3.5835)	(3.4873)	(3.4995)	(3.2101)	(3.1700)	*(3.1644)*	(2.3438)	(2.3436)	(2.3337)	(3.1510)
			0.75	**0.9495**	**0.9495**	**0.9505**	**0.9460**	0.9440	0.9440	0.9045	0.9000	0.8995	0.8665
				(13.5564)	(13.2198)	(13.2140)	*(11.7502)*	(11.5513)	(11.5558)	(7.8625)	(7.8352)	(7.8118)	(7.9320)
			1	**0.9475**	**0.9485**	0.9445	0.9405	0.9415	0.9405	0.9105	0.9050	0.9020	0.8440
				(43.9448)	*(42.4819)*	(42.6108)	(36.3296)	(35.5742)	(35.5211)	(22.0256)	(21.8775)	(21.8207)	(20.0600)
	0.5	1	0.25	**0.9480**	0.9150	0.9255	0.9355	0.9330	0.9300	0.8935	0.8855	0.8815	**0.9975**
				*(0.2260)*	(0.2132)	(0.2156)	(0.2039)	(0.2108)	(0.2036)	(0.1592)	(0.1709)	(0.1629)	(0.4310)
			0.5	0.9410	0.9375	0.9380	0.9305	0.9380	0.9340	0.8895	0.8850	0.8805	0.9400
				(1.1711)	(1.1045)	(1.1167)	(0.9892)	(0.9646)	(0.9605)	(0.6322)	(0.6372)	(0.6284)	(0.8288)
			0.75	**0.9465**	0.9400	0.9445	0.9395	**0.9455**	0.9435	0.8830	0.8870	0.8860	0.8610
				(5.2025)	(4.7248)	(4.7697)	(3.9928)	*(3.7855)*	(3.7703)	(2.1150)	(2.0986)	(2.0744)	(2.0460)
			1	**0.9545**	**0.9485**	**0.9470**	0.9430	**0.9470**	**0.9455**	0.8825	0.8770	0.8750	0.8195
				(16.3088)	(14.9314)	(14.9998)	(11.7841)	(11.1819)	*(11.1794)*	(5.3498)	(5.3624)	(5.3033)	(4.8820)
		2	0.25	**0.9470**	0.9155	0.9290	0.9340	0.9340	0.9315	0.8985	0.8905	0.8840	**0.9990**
				*(0.8859)*	(0.8349)	(0.8453)	(0.7993)	(0.8288)	(0.8014)	(0.6258)	(0.6728)	(0.6414)	(1.7280)
			0.5	**0.9475**	0.9385	0.9385	0.9410	0.9390	0.9380	0.8815	0.8745	0.8740	0.9425
				*(4.4994)*	(4.2041)	(4.2433)	(3.7421)	(3.6338)	(3.6219)	(2.4120)	(2.4183)	(2.3876)	(3.3040)
			0.75	**0.9505**	0.9440	0.9435	0.9440	0.9435	0.9395	0.8910	0.8910	0.8900	0.8590
				*(20.0482)*	(18.2364)	(18.4496)	(15.3255)	(14.6024)	(14.5534)	(8.1464)	(8.1313)	(8.0384)	(8.0500)
			1	0.9405	0.9340	0.9360	0.9285	0.9335	0.9325	0.8910	0.8860	0.8830	0.8220
				(70.7625)	(63.9349)	(63.9721)	(50.3924)	(47.2016)	(47.1224)	(22.4075)	(22.1787)	(21.9509)	(19.2900)
50	0.1	1	0.25	**0.9470**	0.5545	0.6225	0.9440	**0.9475**	0.8815	0.9205	**0.9465**	0.8710	**0.9510**
				*(0.0832)*	(0.0841)	(0.0841)	(0.0799)	(0.1454)	(0.1190)	(0.0707)	(0.1401)	(0.1121)	(0.1967)
			0.5	**0.9495**	0.9275	0.9305	0.9440	**0.9450**	0.9425	0.9100	0.9260	0.9180	0.9280
				(0.4867)	(0.4852)	(0.4858)	(0.4640)	*(0.4781)*	(0.4723)	(0.3998)	(0.4162)	(0.4093)	(0.4759)
			0.75	0.9420	0.9430	0.9435	0.9365	0.9370	0.9380	0.9115	0.9090	0.9095	0.8995
				(1.7662)	(1.7559)	(1.7557)	(1.6697)	(1.6654)	(1.6658)	(1.4010)	(1.4050)	(1.4045)	(1.4040)
			1	0.9410	0.9390	0.9415	0.9410	0.9425	0.9425	0.9240	0.9240	0.9235	0.8805
				(5.1493)	(5.1123)	(5.1115)	(4.8077)	(4.7908)	(4.7900)	(3.8953)	(3.8987)	(3.8970)	(3.7480)
		2	0.25	**0.9545**	0.5900	0.6290	**0.9490**	**0.9560**	0.8995	0.9305	**0.9565**	0.8930	0.9420
				(0.3349)	(0.3370)	(0.3385)	*(0.3219)*	(0.5821)	(0.4775)	(0.2846)	(0.5607)	(0.4497)	(0.7775)
			0.5	**0.9540**	0.9415	**0.9495**	**0.9525**	**0.9585**	**0.9530**	0.9260	0.9365	0.9280	0.9380
				(1.9413)	(1.9350)	(1.9377)	*(1.8490)*	(1.9059)	(1.8818)	(1.5932)	(1.6593)	(1.6323)	(1.9100)
			0.75	**0.9545**	**0.9540**	**0.9555**	**0.9520**	**0.9535**	**0.9535**	0.9225	0.9250	0.9265	0.9025
				(6.9250)	(6.8875)	(6.8851)	(6.5361)	(6.5245)	*(6.5215)*	(5.4790)	(5.4940)	(5.4916)	(5.6770)
			1	**0.9500**	**0.9485**	**0.9500**	0.9440	0.9440	0.9435	0.9255	0.9255	0.9240	0.8980
				(20.4533)	(20.3047)	*(20.2981)*	(19.0203)	(18.9490)	(18.9428)	(15.3698)	(15.3797)	(15.3735)	(14.8200)
	0.3	1	0.25	**0.9515**	0.8330	0.8525	**0.9480**	**0.9535**	0.9290	0.9100	0.9425	0.9090	**0.9970**
				(0.1208)	(0.1189)	(0.1193)	*(0.1163)*	(0.1458)	(0.1329)	(0.1006)	(0.1324)	(0.1190)	(0.3026)
			0.5	**0.9455**	0.9420	**0.9475**	0.9420	**0.9465**	0.9425	0.9090	0.9105	0.9100	**0.9625**
				(0.5785)	(0.5698)	(0.5718)	(0.5462)	*(0.5445)*	(0.5436)	(0.4390)	(0.4415)	(0.4402)	(0.6126)
			0.75	**0.9550**	**0.9545**	**0.9565**	**0.9470**	**0.9485**	**0.9485**	0.9200	0.9180	0.9170	0.9170
				(2.0053)	(1.9717)	(1.9776)	(1.8702)	(1.8528)	*(1.8519)*	(1.4359)	(1.4343)	(1.4309)	(1.5650)
			1	0.9350	0.9350	0.9375	0.9290	0.9295	0.9290	0.9075	0.9075	0.9060	0.8695
				(5.6849)	(5.5999)	(5.5954)	(5.1798)	(5.1501)	(5.1384)	(3.8338)	(3.8485)	(3.8340)	(3.8130)
		2	0.25	**0.9455**	0.8215	0.8490	0.9405	**0.9465**	0.9190	0.9110	0.9245	0.8915	**0.9980**
				*(0.4813)*	(0.4737)	(0.4762)	(0.4630)	(0.5804)	(0.5296)	(0.4006)	(0.5277)	(0.4746)	(1.2140)
			0.5	**0.9520**	**0.9485**	**0.9520**	**0.9485**	**0.9510**	**0.9490**	0.9120	0.9150	0.9115	**0.9565**
				(2.3071)	(2.2695)	(2.2775)	(2.1806)	(2.1713)	*(2.1678)*	(1.7556)	(1.7618)	(1.7559)	(2.3770)
			0.75	**0.9565**	**0.9515**	**0.9530**	**0.9490**	**0.9485**	**0.9480**	0.9100	0.9100	0.9105	0.9115
				(7.8518)	(7.7266)	(7.7310)	(7.2992)	(7.2340)	*(7.2328)*	(5.6277)	(5.6248)	(5.6123)	(6.3210)
			1	**0.9540**	**0.9525**	**0.9515**	**0.9520**	**0.9520**	**0.9510**	0.9185	0.9180	0.9170	0.8700
				(22.3089)	(22.0112)	(22.0740)	(20.3208)	(20.2386)	*(20.2162)*	(14.9722)	(15.0632)	(15.0148)	(15.0800)
	0.5	1	0.25	**0.9520**	0.9210	0.9385	**0.9460**	0.9355	0.9360	0.9140	0.9035	0.9010	**1.0000**
				(0.1518)	(0.1467)	(0.1476)	*(0.1442)*	(0.1495)	(0.1452)	(0.1226)	(0.1295)	(0.1249)	(0.3354)
			0.5	**0.9545**	0.9435	**0.9470**	**0.9485**	**0.9480**	**0.9465**	0.8995	0.8985	0.8965	**0.9525**
				(0.6539)	(0.6384)	(0.6396)	(0.6022)	(0.6029)	*(0.5986)*	(0.4533)	(0.4619)	(0.4562)	(0.6404)
			0.75	0.9420	0.9390	0.9370	0.9400	0.9430	0.9415	0.9090	0.9080	0.9015	0.9040
				(2.3325)	(2.2605)	(2.2604)	(2.0837)	(2.0668)	(2.0585)	(1.4355)	(1.4547)	(1.4383)	(1.5730)
			1	**0.9570**	**0.9465**	**0.9455**	**0.9500**	**0.9480**	**0.9460**	0.9075	0.9085	0.9075	0.8695
				(6.5910)	(6.4267)	(6.4708)	(5.7676)	(5.7452)	*(5.7192)*	(3.7059)	(3.7752)	(3.7346)	(3.6600)
50	0.5	2	0.25	**0.9510**	0.9355	**0.9450**	**0.9460**	**0.9510**	**0.9460**	0.9215	0.9150	0.9115	**0.9990**
				(0.6006)	(0.5828)	(0.5863)	*(0.5714)*	(0.5932)	(0.5763)	(0.4869)	(0.5150)	(0.4971)	(1.3400)
			0.5	**0.9475**	0.9400	**0.9455**	**0.9450**	**0.9460**	0.9435	0.8995	0.8955	0.8945	**0.9595**
				(2.6358)	(2.5585)	(2.5736)	(2.4226)	*(2.4189)*	(2.3996)	(1.8206)	(1.8495)	(1.8268)	(2.6040)
			0.75	**0.9565**	**0.9495**	**0.9530**	**0.9450**	**0.9475**	**0.9475**	0.9065	0.9070	0.9065	0.9070
				(9.0899)	(8.7917)	(8.8439)	(8.1954)	(8.1086)	*(8.0593)*	(5.6316)	(5.7024)	(5.6376)	(6.1990)
			1	**0.9585**	**0.9510**	**0.9535**	**0.9485**	**0.9485**	**0.9480**	0.9080	0.9065	0.9065	0.8715
				(27.5466)	(26.4178)	(26.5151)	(24.0987)	(23.5513)	*(23.4742)*	(15.2586)	(15.3109)	(15.1464)	(14.7300)
70	0.1	1	0.25	**0.9505**	0.5685	0.6120	**0.9490**	**0.9545**	0.8865	0.9260	**0.9505**	0.8780	**0.9700**
				(0.0679)	(0.0685)	(0.0686)	*(0.0661)*	(0.1238)	(0.1005)	(0.0594)	(0.1201)	(0.0957)	(0.1681)
			0.5	0.9430	0.9355	0.9320	0.9420	0.9425	0.9370	0.9170	0.9260	0.9215	0.9465
				(0.3807)	(0.3801)	(0.3803)	(0.3683)	(0.3813)	(0.3754)	(0.3271)	(0.3414)	(0.3358)	(0.4044)
			0.75	**0.9455**	**0.9475**	**0.9455**	0.9435	**0.9450**	0.9435	0.9285	0.9285	0.9295	0.9125
				(1.3891)	(1.3833)	(1.3846)	(1.3331)	*(1.3332)*	(1.3323)	(1.1665)	(1.1716)	(1.1711)	(1.1920)
			1	**0.9530**	**0.9535**	**0.9550**	**0.9505**	**0.9515**	**0.9515**	0.9345	0.9340	0.9335	0.8970
				(3.8672)	(3.8486)	(3.8496)	(3.6632)	(3.6580)	*(3.6579)*	(3.1336)	(3.1417)	(3.1423)	(3.1000)
		2	0.25	0.9420	0.5545	0.5975	0.9380	**0.9575**	0.8835	0.9170	**0.9540**	0.8740	**0.9620**
				(0.2703)	(0.2731)	(0.2723)	(0.2629)	(0.4936)	(0.3994)	(0.2363)	*(0.4793)*	(0.3810)	(0.6732)
			0.5	**0.9525**	0.9280	0.9370	**0.9505**	**0.9510**	0.9440	0.9315	0.9350	0.9265	**0.9580**
				(1.5570)	(1.5541)	(1.5559)	*(1.5047)*	(1.5549)	(1.5334)	(1.3350)	(1.3915)	(1.3695)	(1.6110)
			0.75	0.9430	0.9430	0.9435	0.9425	0.9435	0.9415	0.9205	0.9210	0.9210	0.9205
				(5.5039)	(5.4856)	(5.4879)	(5.2796)	(5.2789)	(5.2777)	(4.6180)	(4.6410)	(4.6389)	(4.8500)
			1	0.9425	0.9400	0.9405	0.9345	0.9350	0.9355	0.9170	0.9175	0.9185	0.8955
				(15.7916)	(15.7195)	(15.7238)	(15.0153)	(15.0011)	(15.0027)	(12.8193)	(12.8654)	(12.8640)	(12.6000)
	0.3	1	0.25	**0.9510**	0.8035	0.8290	**0.9450**	0.9385	0.9075	0.9130	0.9245	0.8855	**0.9980**
				(0.0990)	(0.0978)	(0.0982)	*(0.0963)*	(0.1217)	(0.1105)	(0.0852)	(0.1126)	(0.1008)	(0.2575)
			0.5	**0.9500**	**0.9480**	**0.9500**	**0.9480**	**0.9475**	**0.9465**	0.9175	0.9170	0.9195	**0.9675**
				(0.4462)	(0.4417)	(0.4424)	(0.4286)	(0.4291)	*(0.4279)*	(0.3582)	(0.3612)	(0.3600)	(0.5105)
			0.75	**0.9550**	**0.9515**	**0.9520**	**0.9450**	**0.9480**	**0.9465**	0.9235	0.9195	0.9210	0.9235
				(1.5133)	(1.4925)	(1.4954)	(1.4375)	(1.4279)	(1.4273)	*(1.1694)*	(1.1701)	(1.1680)	(1.3100)
			1	**0.9515**	**0.9515**	**0.9475**	0.9395	0.9420	0.9405	0.9120	0.9190	0.9175	0.8950
				(4.1597)	*(4.1128)*	(4.1209)	(3.8964)	(3.8915)	(3.8835)	(3.0928)	(3.1118)	(3.1027)	(3.1680)
		2	0.25	**0.9490**	0.8125	0.8565	**0.9450**	**0.9460**	0.9205	0.9240	0.9380	0.9005	**0.9975**
				(0.3943)	(0.3899)	(0.3910)	*(0.3839)*	(0.4871)	(0.4414)	(0.3398)	(0.4510)	(0.4033)	(1.0270)
			0.5	**0.9480**	0.9440	**0.9470**	**0.9470**	**0.9475**	**0.9460**	0.9245	0.9195	0.9180	**0.9680**
				(1.7944)	(1.7756)	(1.7789)	(1.7210)	(1.7216)	*(1.7179)*	(1.4438)	(1.4543)	(1.4491)	(2.0400)
			0.75	**0.9465**	**0.9485**	**0.9485**	0.9445	**0.9460**	**0.9450**	0.9185	0.9160	0.9155	0.9245
				(6.0531)	(6.0050)	(6.0125)	(5.7373)	(5.7260)	*(5.7200)*	(4.6957)	(4.7173)	(4.7089)	(5.2490)
			1	**0.9525**	**0.9485**	**0.9535**	**0.9470**	**0.9475**	**0.9460**	0.9155	0.9140	0.9125	0.9030
				(16.6790)	(16.5342)	(16.5201)	(15.5679)	(15.5612)	*(15.5351)*	(12.3975)	(12.4868)	(12.4470)	(12.8000)
	0.5	1	0.25	**0.9455**	0.9315	0.9375	0.9435	**0.9480**	0.9445	0.9190	0.9145	0.9130	**0.9995**
				*(0.1214)*	(0.1188)	(0.1193)	(0.1173)	(0.1215)	(0.1185)	(0.1032)	(0.1083)	(0.1051)	(0.2843)
			0.5	**0.9555**	**0.9455**	**0.9485**	**0.9535**	**0.9560**	**0.9505**	0.9110	0.9080	0.9085	**0.9665**
				(0.5014)	(0.4927)	(0.4934)	(0.4754)	(0.4785)	*(0.4744)*	(0.3787)	(0.3864)	(0.3816)	(0.5469)
			0.75	**0.9470**	0.9395	0.9415	0.9405	0.9375	0.9350	0.8995	0.8925	0.8885	0.9220
				*(1.6158)*	(1.5864)	(1.5898)	(1.5125)	(1.5163)	(1.5076)	(1.1367)	(1.1585)	(1.1460)	(1.3220)
			1	**0.9515**	0.9445	**0.9485**	**0.9495**	**0.9510**	**0.9480**	0.9085	0.9150	0.9130	0.8780
				(4.3643)	(4.2973)	(4.3012)	(3.9679)	(3.9781)	*(3.9531)*	(2.8623)	(2.9176)	(2.8879)	(3.0120)
		2	0.25	**0.9485**	0.9260	0.9400	0.9435	**0.9510**	0.9435	0.9235	0.9260	0.9155	**1.0000**
				(0.4868)	(0.4755)	(0.4782)	(0.4701)	*(0.4863)*	(0.4744)	(0.4132)	(0.4333)	(0.4205)	(1.1360)
			0.5	**0.9470**	0.9385	0.9440	0.9390	0.9410	0.9390	0.9020	0.9015	0.8970	**0.9645**
				*(2.0039)*	(1.9570)	(1.9634)	(1.9038)	(1.9085)	(1.8932)	(1.5102)	(1.5373)	(1.5183)	(2.1770)
			0.75	**0.9510**	**0.9450**	0.9395	**0.9470**	**0.9450**	0.9430	0.9015	0.9065	0.9050	0.9295
				(6.5959)	(6.4775)	(6.4983)	*(6.1463)*	(6.1703)	(6.1311)	(4.6165)	(4.7141)	(4.6601)	(5.2630)
			1	**0.9545**	**0.9485**	**0.9500**	**0.9500**	**0.9495**	**0.9470**	0.9115	0.9110	0.9040	0.8985
				(17.7282)	(17.3594)	(17.3515)	(16.1975)	(16.1722)	*(16.0786)*	(11.6063)	(11.7858)	(11.6622)	(12.1700)
100	0.1	1	0.25	0.9415	0.5510	0.5930	0.9400	0.9430	0.8660	0.9220	0.9410	0.8545	**0.9710**
				(0.0553)	(0.0556)	(0.0556)	(0.0542)	(0.1037)	(0.0833)	(0.0495)	(0.1012)	(0.0803)	*(0.1411)*
			0.5	**0.9510**	0.9305	0.9400	**0.9490**	**0.9505**	**0.9500**	0.9330	0.9355	0.9310	**0.9535**
				(0.3078)	(0.3075)	(0.3077)	*(0.3004)*	(0.3112)	(0.3064)	(0.2728)	(0.2848)	(0.2797)	(0.3375)
			0.75	**0.9450**	0.9445	**0.9460**	**0.9455**	**0.9460**	**0.9470**	0.9305	0.9340	0.9335	0.9280
				(1.0873)	(1.0842)	(1.0845)	*(1.0534)*	(1.0540)	(1.0538)	(0.9505)	(0.9555)	(0.9553)	(1.0000)
			1	**0.9560**	**0.9555**	**0.9555**	**0.9515**	**0.9515**	**0.9515**	0.9385	0.9375	0.9370	0.9215
				(2.9835)	(2.9709)	(2.9718)	(2.8675)	*(2.8645)*	*(2.8645)*	(2.5570)	(2.5654)	(2.5647)	(2.5940)
		2	0.25	**0.9525**	0.5505	0.6070	**0.9485**	**0.9525**	0.8785	0.9240	**0.9500**	0.8670	**0.9685**
				(0.2212)	(0.2225)	(0.2226)	*(0.2171)*	(0.4155)	(0.3344)	(0.1980)	(0.4057)	(0.3213)	(0.5640)
			0.5	**0.9475**	0.9340	0.9325	**0.9455**	**0.9470**	0.9415	0.9350	0.9385	0.9350	**0.9595**
				(1.2256)	(1.2243)	(1.2251)	*(1.1962)*	(1.2397)	(1.2208)	(1.0857)	(1.1348)	(1.1140)	(1.3430)
			0.75	**0.9520**	**0.9520**	**0.9505**	**0.9465**	**0.9437**	**0.9460**	0.9295	0.9350	0.9340	0.9250
				(4.3851)	(4.3720)	(4.3726)	(4.2514)	(4.2501)	(4.2491)	(3.8274)	(3.8439)	(3.8427)	(4.0260)
			1	**0.9475**	**0.9450**	**0.9490**	0.9430	0.9445	0.9435	0.9290	0.9285	0.9285	0.9210
				(11.9827)	*(11.9340)*	(11.9350)	(11.4984)	(11.4810)	(11.4772)	(10.2306)	(10.2612)	(10.2597)	(10.3800)
	0.3	1	0.25	**0.9500**	0.8080	0.8345	**0.9455**	**0.9515**	0.9175	0.9160	0.9390	0.8965	**0.9990**
				(0.0800)	(0.0792)	(0.0795)	*(0.0784)*	(0.1005)	(0.0907)	(0.0706)	(0.0942)	(0.0839)	(0.2153)
			0.5	**0.9465**	**0.9470**	**0.9450**	**0.9455**	**0.9475**	**0.9460**	0.9250	0.9240	0.9205	**0.9745**
				(0.3607)	(0.3582)	(0.3586)	*(0.3501)*	(0.3511)	(0.3502)	(0.3015)	(0.3042)	(0.3033)	(0.4278)
			0.75	**0.9495**	**0.9475**	**0.9495**	**0.9475**	**0.9490**	**0.9485**	0.9280	0.9255	0.9245	0.9325
				(1.1735)	(1.1656)	(1.1673)	(1.1345)	(1.1330)	*(1.1326)*	(0.9612)	(0.9652)	(0.9639)	(1.1020)
			1	**0.9530**	**0.9495**	**0.9520**	**0.9470**	**0.9475**	**0.9465**	0.9200	0.9250	0.9240	0.9325
				(3.1462)	(3.1255)	(3.1314)	*(2.9870)*	(2.9943)	(2.9876)	(2.5045)	(2.5262)	(2.5190)	(2.6840)
		2	0.25	**0.9465**	0.8100	0.8375	**0.9465**	**0.9450**	0.9155	0.9240	0.9345	0.8955	**0.9985**
				(0.3192)	(0.3167)	(0.3174)	*(0.3127)*	(0.4020)	(0.3623)	(0.2825)	(0.3777)	(0.3362)	(0.8609)
			0.5	0.9430	0.9440	0.9415	0.9405	**0.9465**	0.9445	0.9155	0.9185	0.9165	**0.9815**
				(1.4291)	(1.4164)	(1.4196)	(1.3881)	*(1.3909)*	(1.3876)	(1.1972)	(1.2068)	(1.2032)	(1.7100)
			0.75	**0.9480**	0.9445	0.9435	0.9430	0.9415	0.9420	0.9155	0.9165	0.9170	0.9440
				*(4.7165)*	(4.6861)	(4.6901)	(4.5483)	(4.5428)	(4.5396)	(3.8565)	(3.8734)	(3.8670)	(4.4200)
			1	**0.9575**	**0.9555**	**0.9570**	**0.9535**	**0.9550**	**0.9555**	0.9310	0.9345	0.9350	0.9235
				(12.5074)	(12.3930)	(12.3895)	(11.9186)	(11.9000)	*(11.8777)*	(9.9598)	(10.0143)	(9.9831)	(10.6400)
100	0.5	1	0.25	0.9425	0.9235	0.9305	0.9410	0.9380	0.9375	0.9150	0.9135	0.9095	**1.0000**
				(0.0987)	(0.0972)	(0.0975)	(0.0964)	(0.0992)	(0.0973)	(0.0866)	(0.0901)	(0.0881)	*(0.2391)*
			0.5	**0.9460**	0.9380	0.9405	**0.9460**	0.9425	0.9410	0.9145	0.9140	0.9120	**0.9730**
				(0.3977)	(0.3927)	(0.3943)	*(0.3833)*	(0.3877)	(0.3845)	(0.3176)	(0.3250)	(0.3214)	(0.4559)
			0.75	**0.9465**	0.9375	0.9425	0.9440	0.9410	0.9405	0.8945	0.8980	0.8970	0.9320
				*(1.1987)*	(1.1858)	(1.1855)	(1.1395)	(1.1496)	(1.1423)	(0.9102)	(0.9316)	(0.9207)	(1.1110)
			1	**0.9510**	**0.9465**	**0.9470**	**0.9475**	**0.9480**	**0.9490**	0.9140	0.9160	0.9125	0.8945
				(3.2068)	(3.1582)	(3.1690)	(3.0107)	(3.0277)	*(3.0067)*	(2.3287)	(2.3731)	(2.3499)	(2.5490)
		2	0.25	0.9430	0.9260	0.9355	0.9385	0.9430	0.9370	0.9210	0.9190	0.9160	**1.0000**
				(0.3954)	(0.3893)	(0.3908)	(0.3867)	(0.3974)	(0.3902)	(0.3477)	(0.3610)	(0.3532)	*(0.9579)*
			0.5	**0.9465**	0.9385	0.9420	0.9440	**0.9450**	0.9430	0.9105	0.9075	0.9070	**0.9775**
				(1.5561)	(1.5344)	(1.5369)	(1.4986)	*(1.5135)*	(1.5001)	(1.2423)	(1.2697)	(1.2541)	(1.8350)
			0.75	**0.9545**	**0.9470**	0.9445	**0.9545**	**0.9510**	**0.9495**	0.9170	0.9180	0.9150	0.9275
				(4.8595)	(4.8071)	(4.8234)	(4.6301)	(4.6799)	*(4.6454)*	(3.6927)	(3.7840)	(3.7393)	(4.4050)
			1	**0.9460**	0.9395	0.9415	0.9430	**0.9490**	**0.9475**	0.9145	0.9160	0.9140	0.9035
				(13.0948)	(12.9117)	(12.9682)	(12.2906)	(12.3802)	*(12.2868)*	(9.5377)	(9.7308)	(9.6352)	(10.1300)

**Note:**

The coverage probabilities close to or greater than the nominal confidence level of 0.95 are in bold (values of 0.9450 and above will be rounded to 0.95), and the shortest average widths are in italics.

However, it was observed that as sample sizes increased, the widths of all confidence intervals decreased, and the CP of the NA method increased. Conversely, an increase in the shape parameter, scale parameter, and probabilities of zeros widened the widths for all confidence intervals. In summary, the simulation study showed that GFCI-2 performed the best for small sample sizes ( $n$ = 15, 20, 30), GFCI-3 performed the best for medium sample sizes ( $n$ = 50, 70), and GFCI-1 stood out for large sample sizes ( $n$ = 100). These methods met both CP and AW criteria.

### An empirical application

**Figure 3:**
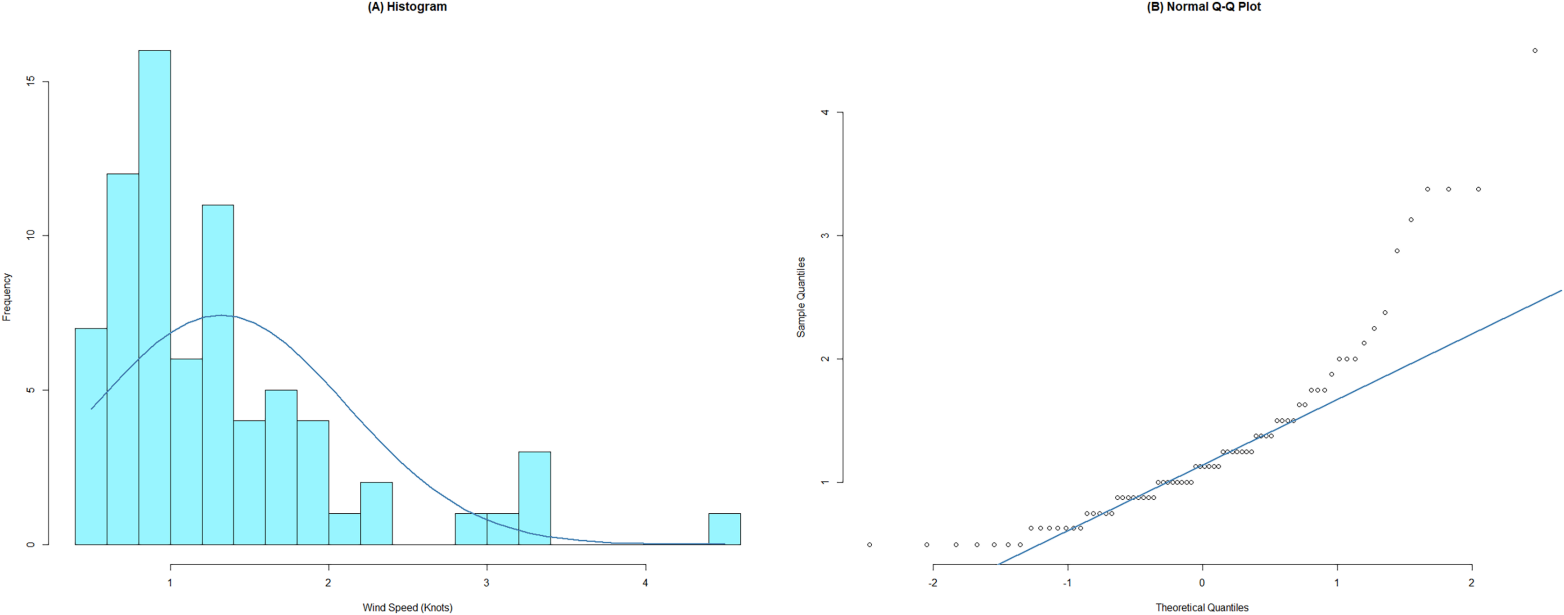
The (A) histogram and (B) normal Q-Q plot of wind speed data from October to December 2023 in Prachin Buri province, Thailand.

**Figure 4:**
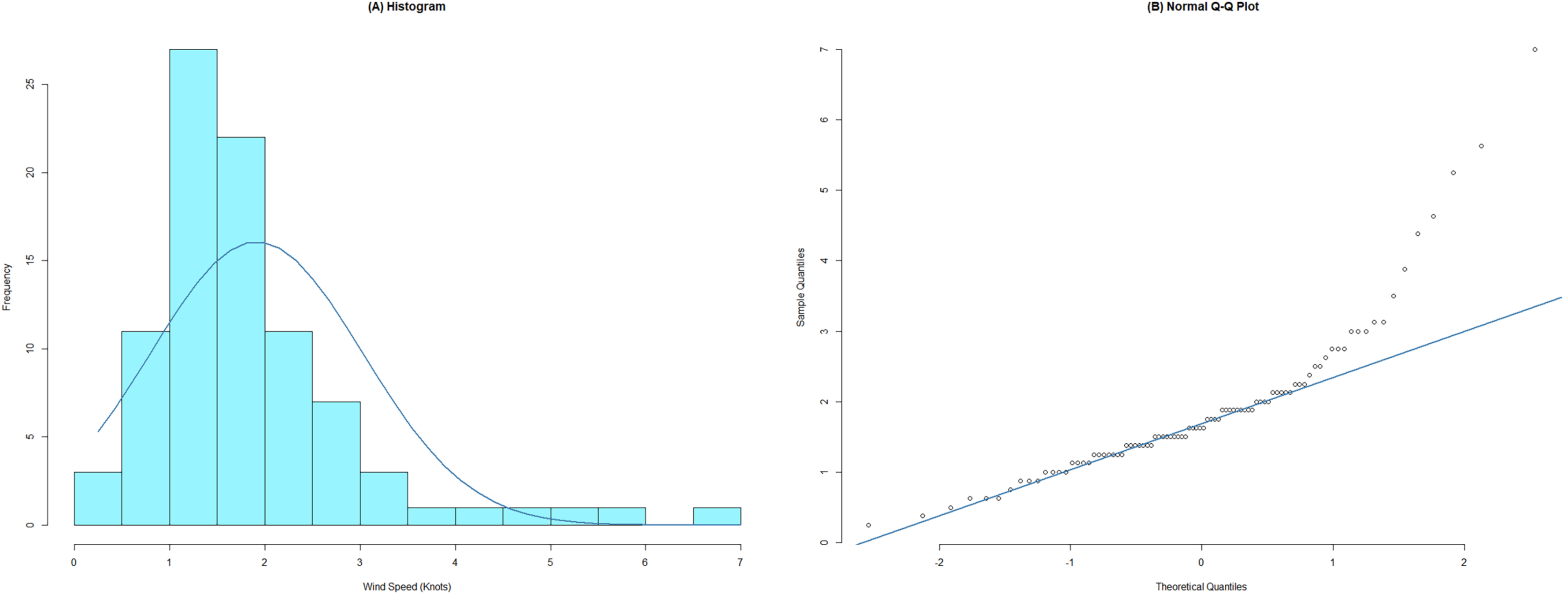
The (A) histogram and (B) normal Q-Q plot of wind speed data from October to December 2023 in Rayong province, Thailand.

Every year, Thailand faces challenges with PM2.5 due to fluctuating weather conditions exacerbated by combustion and industrial activities. PM2.5 poses significant health risks, especially in industrial areas such as Prachin Buri and Rayong provinces, located in the eastern part of Thailand. These provinces recorded the highest levels in 2023, with an AQI of 207 (Thai Pollution Control Department, http://air4thai.pcd.go.th/webV3/#/History). Amnuaylojaroen, Kaewkanchanawong & Panpeng (2023) indicate an inverse relationship between wind speed and PM2.5, emphasizing the importance of wind speed in reducing pollution levels. Based on Mohammadi, Alavi & McGowan (2017), which assessed wind speed at ten stations in Canada using AIC and BIC, the wind speed data showed a better fit to the BirSau distribution compared to nine other models (Rayleigh, Inverse Gaussian, Logistic, Log-Logistic, Normal, Log-Normal, Weibull, Generalized Extreme Value, and Nakagami). Therefore, BirSau distribution is utilized to estimate wind speed and power distributions. Since previous studies using the BirSau distribution (Puggard, Niwitpong & Niwitpong, 2022a, 2022b) focused only on wind speeds greater than zero, the delta-BirSau distribution is more suitable when the wind speed data includes zero values. Therefore, this study is interested in estimating the confidence interval for the variance of wind speeds that include zero values using the delta-BirSau distribution. This highlights the importance of accurately estimating wind speed, particularly in industrial areas. To achieve this, we analyze daily wind speed data from October to December 2023 in industrial settings. Specifically, Prachin Buri and Rayong provinces, Thailand (Thai Meteorological Department Automatic Weather System, https://www.tmd.go.th/service/tmdData), employing ten distinct methods outlined in Table 2 to establish confidence intervals for the wind speed data. In our study, the preprocessing of raw data involves the following key steps, starting with selecting the provinces of interest: First, we collected wind speed data, including zero values, from the website of the Meteorological Department. The raw data was then divided into two categories: data where wind speed is greater than zero and data where wind speed is zero. Next, we performed parameter estimation. For the wind speed data greater than zero, we estimated the parameters $\alpha$ and $\beta$. For the zero wind speed data, we estimated the parameter $\delta$. Following this, we used the estimated parameters to calculate the variance of the wind speed data. Finally, we calculated the confidence intervals for the variance at a 95% confidence level to assess the reliability of our variance estimates. For wind speed data, the parameter $\alpha$ indicates the skewness of the distribution, reflecting the tendency for unusually high wind speeds. The parameter $\beta$ represents the scale and spread of wind speeds in the area, capturing the mean and variance. The parameter $\delta$ shows the proportion of zero values in the dataset, with a higher $\delta$ value indicating more zero values. For wind speed data from Prachin Buri province, there were 92 measurements, consisting of 74 positive values and 18 zeroes. Similarly, there were 92 measurements from Rayong Province, with 90 positives and two zeroes. The histogram and normal Q-Q plot of this data is presented in Figs. 3 and 4. The histogram indicates that the data follow a right-skewed distribution. At the same time, in the Q-Q plot, it is evident that the wind speed data from both provinces does not follow a normal distribution, as all the data points do not lie in a straight line.

**Table 2:** The daily wind speed data from Prachin Buri and Rayong, Thailand.

Provinces	Wind speed (Knots)									
Prachin Buri	1.00	0.00	0.63	0.00	0.00	2.00	1.00	1.50	0.88	3.13
	0.00	1.13	0.88	1.00	0.50	1.25	2.00	1.50	1.13	4.50
	0.00	1.25	0.63	0.75	0.63	3.38	1.38	0.88	0.88	3.38
	0.75	0.00	1.38	1.38	0.50	3.38	2.13	1.00	0.50	1.63
	1.25	0.00	0.50	1.75	1.00	2.88	2.00	2.25	0.00	2.38
	0.00	0.50	1.13	1.50	1.25	1.25	1.00	0.75	1.75	1.38
	0.00	1.88	0.63	0.00	1.13	0.63	0.00	0.00	1.63	0.88
	1.00	0.88	1.13	0.00	0.88	0.75	1.75	0.50	0.63	1.25
	0.75	0.50	1.00	0.88	1.50	0.00	1.25	0.00	1.13	0.63
	0.00	0.00								
Rayong	1.63	0.00	1.00	1.50	1.25	1.50	2.75	3.13	1.50	2.38
	1.13	1.00	1.88	0.50	1.50	2.25	2.50	2.00	1.88	5.25
	1.63	0.88	1.50	1.50	0.38	4.38	1.25	1.38	1.88	3.88
	0.00	0.63	1.63	1.25	1.00	3.00	3.00	1.38	1.13	5.63
	1.63	0.75	1.25	1.88	1.25	2.50	2.13	3.00	1.75	3.50
	7.00	1.88	1.38	2.13	0.63	1.88	2.00	1.50	1.75	2.13
	4.63	1.50	0.88	1.75	0.25	2.75	1.25	1.88	1.38	2.63
	3.13	0.63	1.00	1.13	1.88	2.00	2.25	1.38	2.75	1.13
	1.25	1.38	2.13	2.00	2.13	1.88	1.50	1.63	0.88	2.25
	1.38	1.75								

As the wind speed data consistently consist of zero and positive values, we tested the distributions of positive wind speed data to ascertain if they adhere to BirSau distributions. This assessment used the AIC and BIC. According to Vrieze (2012), when comparing models with continuous and categorical latent variables, it is preferable to determine fit based on a likelihood function that considers higher-order moments of the data. AIC and BIC are helpful because they allow for comparing non-nested models, even when the likelihood functions differ. Therefore, the BirSau distribution with the smallest AIC and BIC values emerged as the best fit for the positive wind speed datasets, as reported in Table 3. Although various distributions, such as the Weibull distribution (Hizoune et al., 2024; Aljeddani & Mohammed, 2023; Kurniawan, 2023), have been used to analyze wind speed data, it is noteworthy that the Weibull distribution provides lower AIC and BIC values for this dataset compared to the BirSau distribution. Therefore, the delta-BirSau distribution is more suitable for the wind speed data we studied. We estimated the parameters $\alpha$, $\beta$, and $\delta$ using the MLE method, with summary statistics derived for the wind speed dataset originating from Prachin Buri province, including values such as ${n_1} = 92$, ${n_{1(0)}} = 18$, ${n_{1(1)}} = 74$, ${\hat \delta _1} = 0.1957$, ${\hat \alpha _1} = 0.5772$, ${\hat \beta _1} = 1.4072$, and ${\hat \tau _1} = 1.1758.$ Correspondingly, summary statistics for the dataset from Rayong province are denoted as ${n_2} = 92$, ${n_{2(0)}} = 2$, ${n_{2(1)}} = 90$, ${\hat \delta _2} = 0.0217$, ${\hat \alpha _2} = 0.6680$, ${\hat \beta _2} = 1.2008$, and ${\hat \tau _2} = 1.0267.$ The calculated two-sided confidence intervals for $\tau$ can be found in Tables 4 and 5.

**Table 3:** AIC and BIC values for fitting the positive wind speed data from Prachin Buri and Rayong, Thailand.

Provinces	Models	Weibull	BirSau
Prachin Buri	AIC	155.5196	137.9650
	BIC	160.1277	144.8770
Rayong	AIC	252.8752	245.8270
	BIC	257.8748	253.3264

**Table 4:** The 95% confidence intervals for the variance of wind speed data for the Prachin Buri province.

Methods	Confidence intervals for $\boldsymbol \tau_1$		Width
	Lower	Upper	
GCI1	0.9952	1.5560	0.5608
GCI2	0.9838	1.5440	0.5602
GCI3	1.0087	1.5670	0.5583
GFCI1	1.0075	1.5390	0.5315
GFCI2	1.0088	1.5530	0.5442
GFCI3	1.0068	1.5470	0.5402
BCI1	1.0175	1.4601	0.4426
BCI2	1.0276	1.4549	0.4273
BCI3	1.0188	1.4696	0.4508
NA	0.8797	1.5020	0.6223

**Table 5:** The 95% confidence intervals for the variance of wind speed data for the Rayong province.

Methods	Confidence intervals for $\boldsymbol \tau_2$		Width
	Lower	Upper	
GCI1	0.9134	2.1880	1.2750
GCI2	0.9347	2.2110	1.2770
GCI3	0.9153	2.1910	1.2760
GFCI1	0.9030	2.0460	1.1420
GFCI2	0.9231	2.0670	1.1440
GFCI3	0.9020	2.0440	1.1430
BCI1	0.8823	1.8990	1.0160
BCI2	0.9050	1.9190	1.0140
BCI3	0.8843	1.9000	1.0160
NA	0.7348	1.894	1.1590

From the wind speed data of Prachin Buri province, when compared with the parameters specified in the data simulation, it was found that with a sample size of $n = 100$, parameters $\delta = 0.1$, $\alpha = 0.5$, and $\beta = 2$, and for Rayong province, with $n = 100$, parameters $\delta = 0.1$, $\alpha = 0.5$, and $\beta = 1$, from Table 1. There are consistent study results: the GCI-1,2,3, GFCI-1,2,3, and NA methods obtained CPs close to the nominal confidence level of 0.95, but the BCI-1,2 and 3 methods are below the nominal confidence level. Therefore, the BCI-1,2 and 3 are not considered. Notably, GFCI-1, utilizing the shortest width method, outperformed GCI-1,2,3, GFCI-1,2 and NA. Thus, for constructing the confidence interval for the variance of wind speed data from October to December 2023 in Prachin Buri and Rayong provinces, Thailand, the GFCI-1 method is recommended.

The proposed method is the most accurate estimation method for wind speed data because it uses statistical techniques and is easy to calculate. Additionally, there are ready-made functions in the R statistics software that make it convenient for users to use efficiently.

## Discussion

Understanding wind speed variability is crucial for assessing how PM2.5 levels disperse. Research by Amnuaylojaroen, Kaewkanchanawong & Panpeng (2023) revealed that increased wind speed correlates with decreased PM2.5 concentrations, demonstrating the impact of wind speed on reducing PM2.5 levels. Therefore, accurately measuring wind speed fluctuations is vital for effective air quality assessment and PM2.5 management. This study aims to estimate the confidence intervals for wind speed variance to address these uncertainties.

The study by Puggard, Niwitpong & Niwitpong (2022a) on confidence intervals for the variance and difference of variances of the BirSau distribution found that the GCI produced a CP greater than the nominal confidence level of 0.95. In contrast, the BCI resulted in a CP less than nominal confidence level of 0.95. These findings are consistent with the results of this work. However, while the BirSau distribution is suitable for data with values greater than zero, the delta-BirSau distribution is recommended for data that includes values of zero.

Thus, the objective of this research was to estimate the confidence interval of the variance of the delta-BirSau distribution utilizing the GCI-1,2,3, GFCI-1,2,3, BCI-1,2,3, and NA methods. The simulation results indicate that the CPs of GCI-1,2,3, and GFCI-1,2,3 were consistently greater than or closed to the nominal confidence level. This is consistent with previous research from La-Ongkaew, Niwitpong & Niwitpong (2021), Maneerat, Nakjai & Niwitpong (2022), and Thangjai, Niwitpong & Niwitpong (2024). In the results of estimating variance confidence intervals using various methods, it was found that both the NA method and the method based on BCI are less computationally demanding and provide shorter AW compared to other methods. However, these methods exhibited a CP less than the nominal confidence level of 0.95. On the other hand, the method based on GFCI demonstrated better accuracy than the method based on GCI, although it required more computational time. However, considering AW together, we find that the GFCI-2 method performs well for small sample groups. For medium-sized samples, the GFCI-3 method is the most appropriate choice. Finally, for large samples, the GFCI-1 method yields the best results.

This study estimates the confidence interval for the variance of the delta-BirSau distribution. Future research could expand this approach to incorporate simultaneous confidence intervals for all pairwise differences between the variances of multiple delta-BirSau distributions. Moreover, the Bayesian credible interval method could be examined to evaluate its effectiveness in estimating these confidence intervals.

## Conclusions

We recommend creating a confidence interval to estimate the variance of the delta-BirSau distribution. This interval is formed through different methods, such as GCI-1,2,3, BCI-1,2,3, GFCI-1,2,3, and NA. To evaluate how well they work, we conducted a numerical study using Monte Carlo simulation, analyzing their CPs and AWs. The simulation results indicate that the CPs of GCI-1,2,3 and GFCI-1,2,3 were greater than or close to the nominal confidence level. However, for small sample sizes, GCI-1 and 3 were under the nominal confidence level. Considering the AW value as well, it is found that GFCI-2 performed the best for small sample sizes, GFCI-3 excelled for medium sample sizes, and GFCI-1 stood out for large sample sizes, making it suitable for constructing the confidence interval for the variance of the delta-BirSau distribution. In contrast, the CPs of BCI-1,2,3 and NA were under the nominal confidence level, rendering them inappropriate solutions for this scenario. Furthermore, when applying these methods to analyze wind speed datasets from Prachin Buri and Rayong provinces in Thailand’s industrial area, the outcomes align with those obtained from the simulation study. The GFCI-1 method is the most suitable for estimating wind speed when the data set is large. The GFCI-3 and GFCI-2 methods are most appropriate for medium and small data sets.

## Supplemental Information

10.7717/peerj.18272/supp-1 Supplemental Information 1 Code R for Computing the Coverage Probabilities and Expected Length.

10.7717/peerj.18272/supp-2 Supplemental Information 2 The daily average wind speed data (Knots) from October to December 2023 in Prachin Buri Province. First column: Date, Second Column: Wind Speed

10.7717/peerj.18272/supp-3 Supplemental Information 3 The daily average wind speed data (Knots) from October to December 2023 in Rayoung Province. First column: Date, Second Column: Wind Speed

## Additional Information and Declarations

### Data Availability

The following information was supplied regarding data availability: The raw measurements are available in the Supplemental Files.
